# The benefit and risk of addition of chemotherapy to EGFR tyrosine kinase inhibitors for EGFR-positive non-small cell lung cancer patients with brain metastases: a meta-analysis based on randomized controlled trials

**DOI:** 10.3389/fonc.2024.1448336

**Published:** 2024-10-21

**Authors:** Zhigang Chen, Xiang Fu, Lingping Zhu, Xiurong Wen, Shihao Zhang

**Affiliations:** ^1^ Department of Oncology, Shangrao People’s Hospital, Shangrao, China; ^2^ Department of Respiratory and Critical Care Medicine, Ganzhou People’s Hospital, Ganzhou, China

**Keywords:** EGFR, tyrosine kinase inhibitors, chemotherapy, non-small cell lung cancer, brain metastases, meta-analysis

## Abstract

**Background:**

Combining epidermal growth factor receptor (EGFR) tyrosine kinase inhibitors (TKIs) with chemotherapy (ETC) offers more advantages for patients with EGFR-positive non-small cell lung cancer (NSCLC) than using EGFR TKIs alone (ET). However, whether this conclusion applies to patients with brain metastases (BM) remains controversial. This meta-analysis was performed to evaluate the benefits and risks of the two groups.

**Methods:**

Six databases were systematically searched for relevant literatures comparing ETC versus ET in treating EGFR-positive NSCLC patients with BM. The primary outcome assessed was overall survival (OS), while secondary outcomes included progression-free survival (PFS), and central nervous system (CNS)-PFS, responses, progression status and safety.

**Results:**

Seven studies based on five randomized clinical trials with 550 patients were included. The ETC group exhibited better OS (hazard ratio [HR]: 0.64 [0.48, 0.87]), PFS (HR: 0.42 [0.34, 0.52]), and CNS-PFS (HR: 0.42 [0.31, 0.57]). The benefits in survival for OS, PFS, and CNS-PFS were validated in nearly all subgroups. Meanwhile, the overall objective response rate (ORR) (risk ratio [RR]: 1.25 [1.02, 1.52]) and CNS-ORR (RR: 1.19 [0.93, 1.51]) also tended to favor the ETC group. However, the addition of chemotherapy also brought about more grade 3-5/serious adverse events (AEs). The top five grade 3-5 AEs in the ETC group were alanine aminotransferase increase (11.25%), neutropenia (7.5%), nausea (7.5%), anorexia (5%), and diarrhea (5%).

**Conclusions:**

ETC appears to be better than ET in treating EGFR-positive NSCLC patients with BM, with better OS, PFS, CNS-PFS, and responses. However, its poorer safety profile also needs to be taken into consideration.

**Systematic review registration:**

https://www.crd.york.ac.uk/PROSPERO/, identifier CRD42024551073.

## Introduction

Lung cancer is the foremost cause of both incidence and mortality among malignant tumors globally, with non-small cell lung cancer (NSCLC) making up about 90% of cases (1). Epidermal growth factor receptor (EGFR) mutations are the most common type among NSCLC cases, occurring in approximately 15% of Western NSCLC patients and 30-40% of Asian patients (2). For advanced EGFR-positive NSCLC, EGFR tyrosine kinase inhibitors (TKIs) significantly extend progression-free survival (PFS) and overall survival (OS) compared to traditional chemotherapy, while reducing the occurrence of adverse events (AEs) (3). The combination of chemotherapy with EGFR-TKIs (ETC) further improves patient outcomes (4). However, there remains clinical debate regarding whether this conclusion applies to EGFR-positive NSCLC patients with brain metastases (BM).

The National Comprehensive Cancer Network (NCCN) and the European Society for Medical Oncology (ESMO) guidelines recommended both EGFR-TKI alone (ET) and ETC as first-line treatments for EGFR-positive NSCLC patients with BM (5, 6). Studies by Lou et al. and Hou et al. had demonstrated that ETC significantly improves patients’ OS and PFS (7, 8). Janne et al. also reported that ETC significantly enhances patients’ central nervous system (CNS) PFS (9). However, study by Miyauchi et al. indicated that ETC did not improve the OS of EGFR-positive NSCLC patients with BM and significantly increases the occurrence of AEs (10).

Addressing the clinical controversy outlined above, this meta-analysis compared the efficacy and safety of ETC and ET treatments in EGFR-positive NSCLC patients with BM.

## Materials and methods

### Selection criteria

Inclusion criteria: (1) Population: EGFR-positive NSCLC patients with BM; (2) Intervention and comparison: ETC versus ET; (3) Outcomes: survival, responses, progression status, and safety; (4) Study design: Randomized clinical trial (RCT).

Exclusion criteria: (1) Case reports, reviews, or meta-analyses; (2) Animal studies; (3) Studies with inaccessible full-text or from which useful data cannot be extracted.

### Search strategy

A computerized search was conducted in PubMed, Scopus, EMBASE, ScienceDirect, Cochrane Library, and Web of Science, covering studies published up to August 27, 2024, that compared ETC and ET in treating EGFR-positive NSCLC patients with BM. The English search terms used were: “EGFR,” “Chemotherapy,” “Lung cancer,” and “Randomized” (**Supplementary Table S1**).

### Data extraction

After independently screening the literature and extracting data, two researchers conducted a cross-check. The extracted data included baseline characteristics of studies (study design, number of patients, etc.), survival outcomes (OS, PFS, CNS-PFS, etc.), responses (ORR, DCR, etc.), progression status (total progression, CNS progression, etc.), and safety indicators (Total AEs, grade 3-5 AEs, etc. AEs were graded according to the National Cancer Institute Common Terminology Criteria for Adverse Events [NCI-CTCAE], version 4.0/5.0) (11, 12). In instances of discrepancies, a third researcher was consulted to make a decision.

### Outcome assessments

The survival rates of PFS, OS, and CNS-PFS were analyzed at 6 to 60 months. Subgroup analyses of PFS, OS, and CNS-PFS were also conducted according to age, sex, ECOG PS, EGFR mutation type, extracranial metastases, and EGFR TKIs.

### Quality assessment

The five-point Jadad scale was used to assess the quality of RCTs, which evaluates randomization, blinding, and patient accountability. Studies with scores of 3 points or higher were considered to be of high quality (13).

The Grades of Recommendations Assessment, Development, and Evaluation (GRADE) system was employed to evaluate the evidence categories of the results, considering five aspects: imprecision, risk of bias, indirectness, inconsistency, and publication bias. The evidence was divided into four categories: very low, low, moderate, and high (14).

## Statistical analysis

The effect measures used included the risk ratio (RR) for binary data and the hazard ratio (HR) for survival data. All effect sizes were presented with 95% confidence intervals (CI). Prior to combining the effect sizes, a test for heterogeneity should be conducted. Heterogeneity among included studies will be assessed using the default Chi-square test. If the p-value is less than 0.1 and the *I* ^2^ statistic is more than 50%, indicating significant heterogeneity. A fixed-effect model will be applied for data analysis if heterogeneity is non-significant. Otherwise, a random-effects model will be used. Funnel plots, Egger’s test, and Begg’s test were conducted to assess publication bias (15–17). REVMAN 5.3 and STATA 12.0 were used for data analysis. This study was conducted following the PRISMA guidelines and registered in PROSPERO (ID: CRD42024551073) (**Supplementary Table S2**).

## Results

### Search results

**Figure 1:**
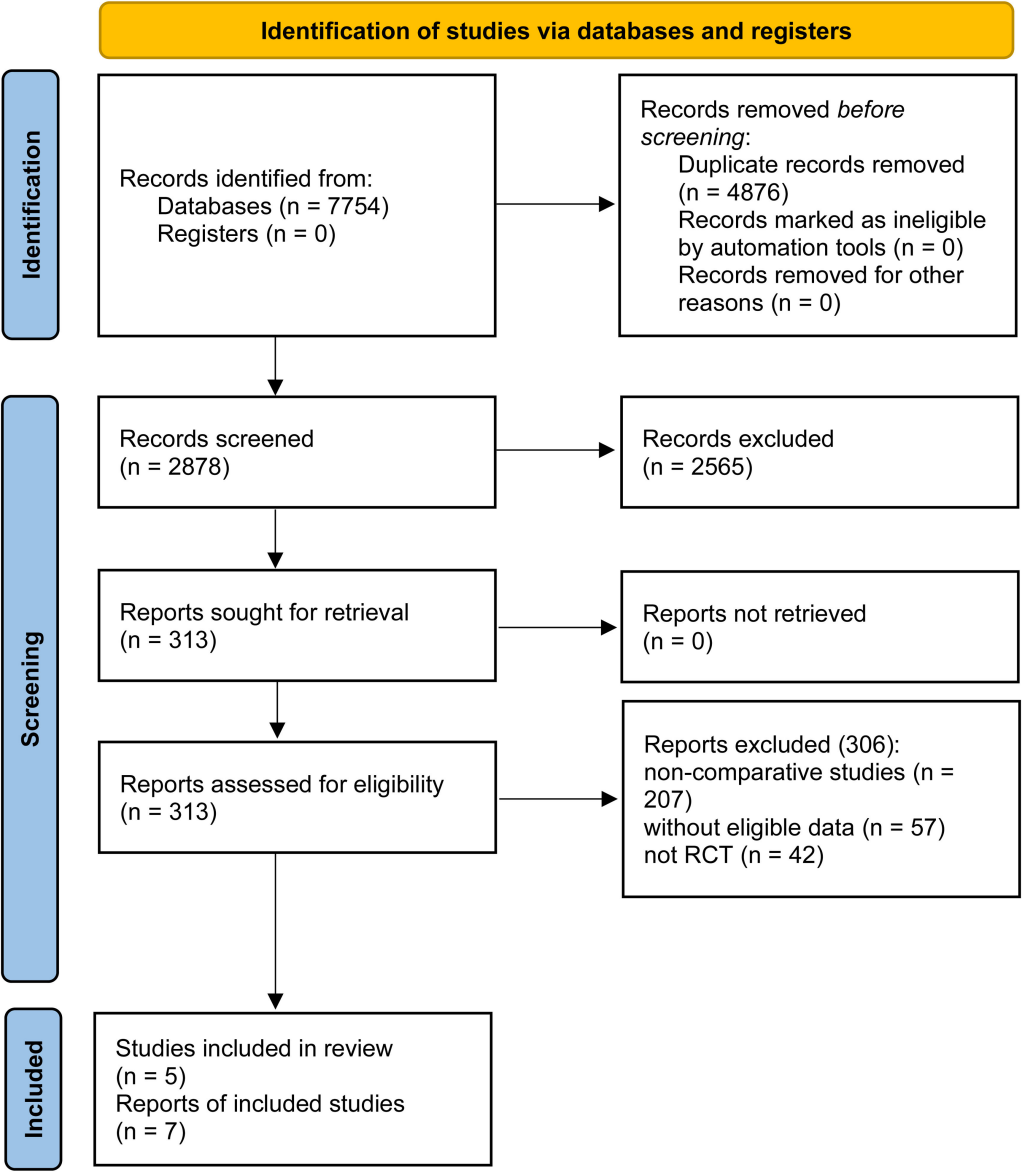
Flow chart.

Seven studies based on 5 RCTs were included (274 patients were in the ETC group, while 276 were in the ET group) (**Figure 1**) (7–10, 18–20). **Table 1** detailed the baseline characteristics of 5 RCTs. Four RCTs (7, 8, 10, 19, 20) were conducted in Asia and another one (9, 18) was global multicenter study. According to the quality assessment, all studies were of medium to high quality (**Supplementary Table S3**, **Supplementary Figure S1**). The quality of evidence for all results, as per the GRADE system, ranged from medium to high (**Supplementary Table S4**).

**Table 1:** Baseline characteristics of the included studies.

Study	Phase	Country	Groups	Patients	Sex (M/F)	Age (Mean, year)	Histologic type (Adeno/Others)	EGFR TKI	Outcomes assessed	Follow up (months)
NCT04035486(FLAURA2, 2020.06-2021.12)										
Janne 2024 (9), Planchard 2023 (18)	III	Global multicenter	ETC	118	36/82	60	118/0	Osimertinib	Survival, Responses, Progression Status,AEs	22
			ET	104	38/66	61	104/0			24
NCT01951469(GAP BRAIN, 2016.01-2021.08)										
Hou 2023 (8)	III	China	ETC	80	36/44	55	76/4	Gefitinib	Survival, Responses, Progression Status,AEs	21
			ET	81	38/43	56	77/4			21
UMIN000006340(NEJ009, 2011.10-2015.09)										
Miyauchi 2022 (10), Hosomi 2020 (19)	III	Japan	ETC	38	–	64	38/0	Gefitinib	Survival	84
			ET	50	–	65	50/0			84
NCT02148380(2011.04-2015.12)										
Lou 2022 (7)	II	China	ETC	8	–	–	8/0	Gefitinib	Survival	–
			ET	7	–	–	7/0			–
CTRI/2016/08/007149(2016.08-2018.08)										
Noronha 2020 (20)	III	India	ETC	30	–	54	30/0	Gefitinib	Survival	17
			ET	34	–	56	34/0			17

EGFR, Epidermal growth factor receptor; ET, EGFR tyrosine kinase inhibitors alone; ETC, EGFR tyrosine kinase inhibitors in combination of chemotherapy; M/F, Male/Female; TKIs, Tyrosine kinase inhibitors.

### Survival

**Figure 2:**
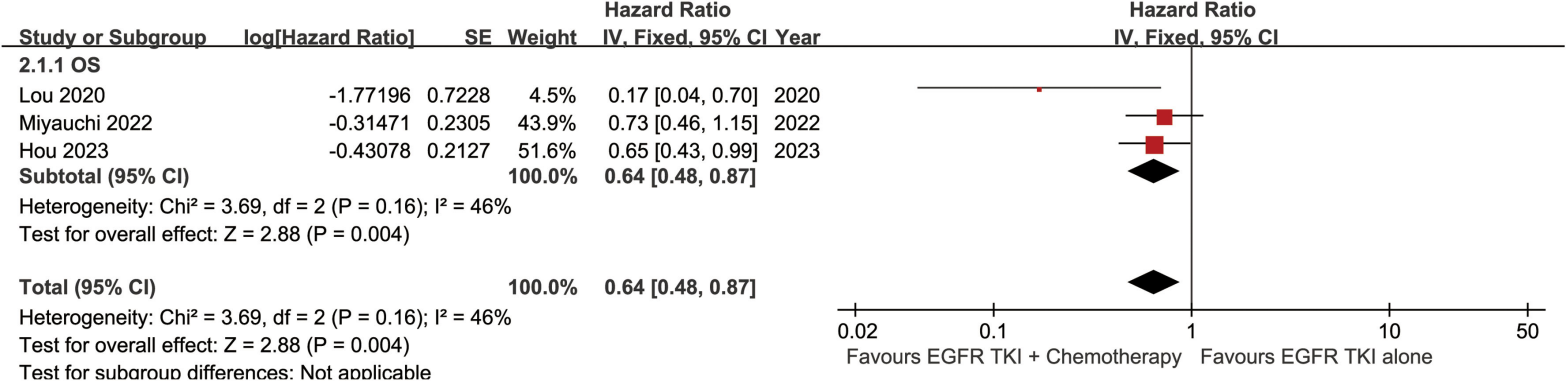
Forest plots of overall survival associated with ETC versus ET.

**Figure 3:**
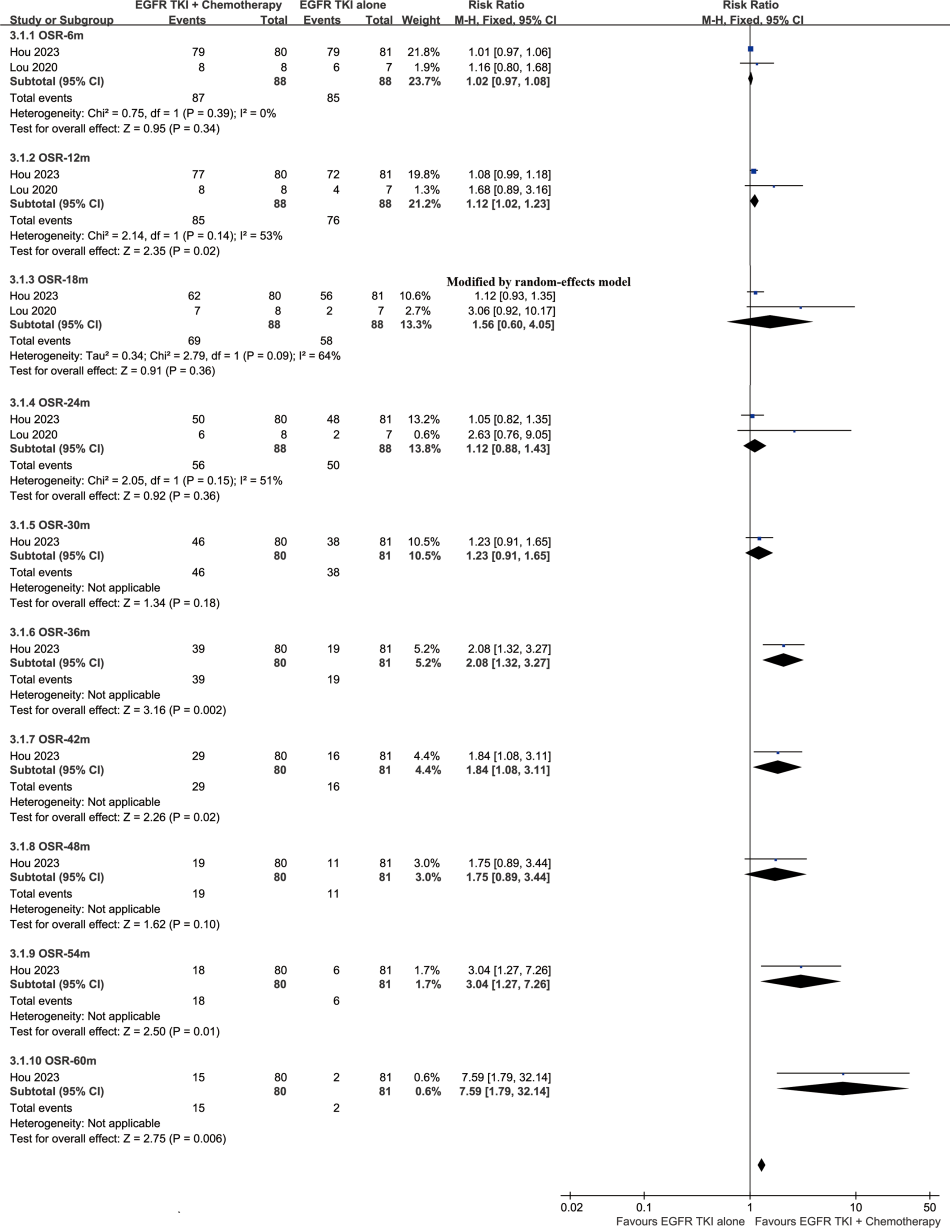
Forest plots of OSR at 6-60 months associated with ETC versus ET.

The OS was better in the ETC group (HR: 0.64 [0.48, 0.87]) (**Figure 2**). The overall survival rate (OSR) also tended to favor the ETC group at 12 to 60 months (**Figure 3**).

**Figure 4:**
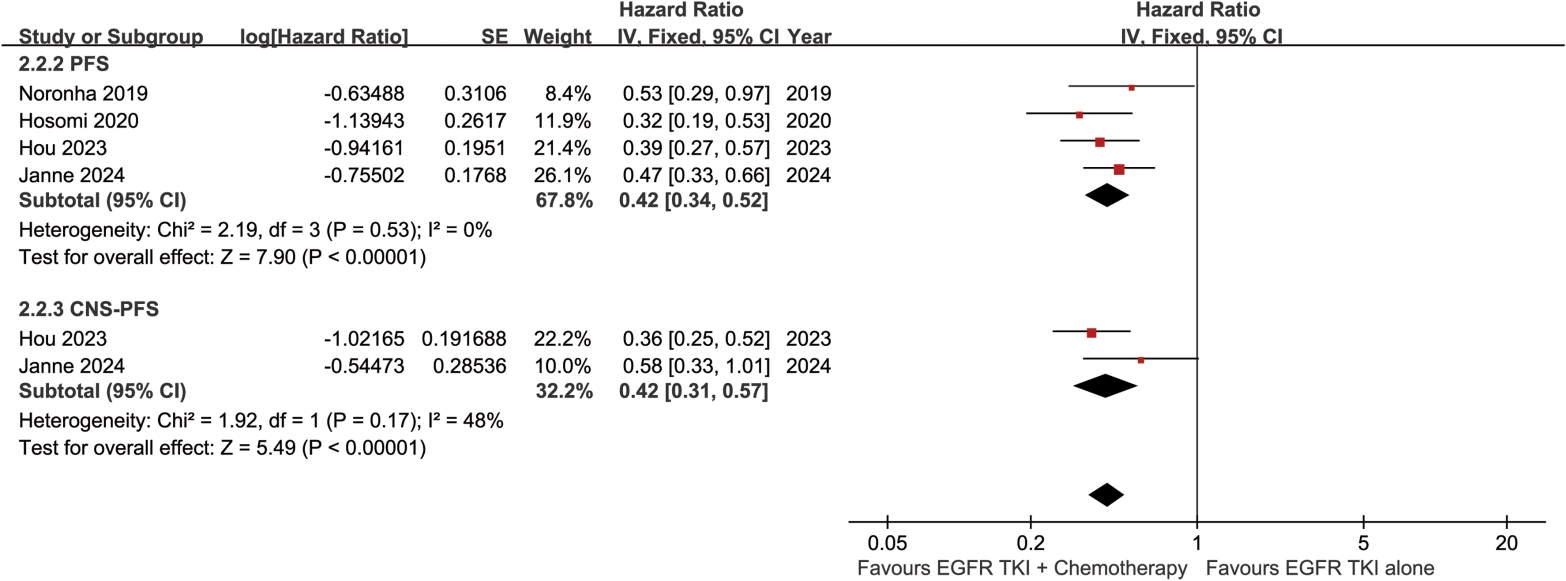
Forest plots of progression-free survival, and CNS-progression-free survival associated with ETC versus ET.

**Figure 5:**
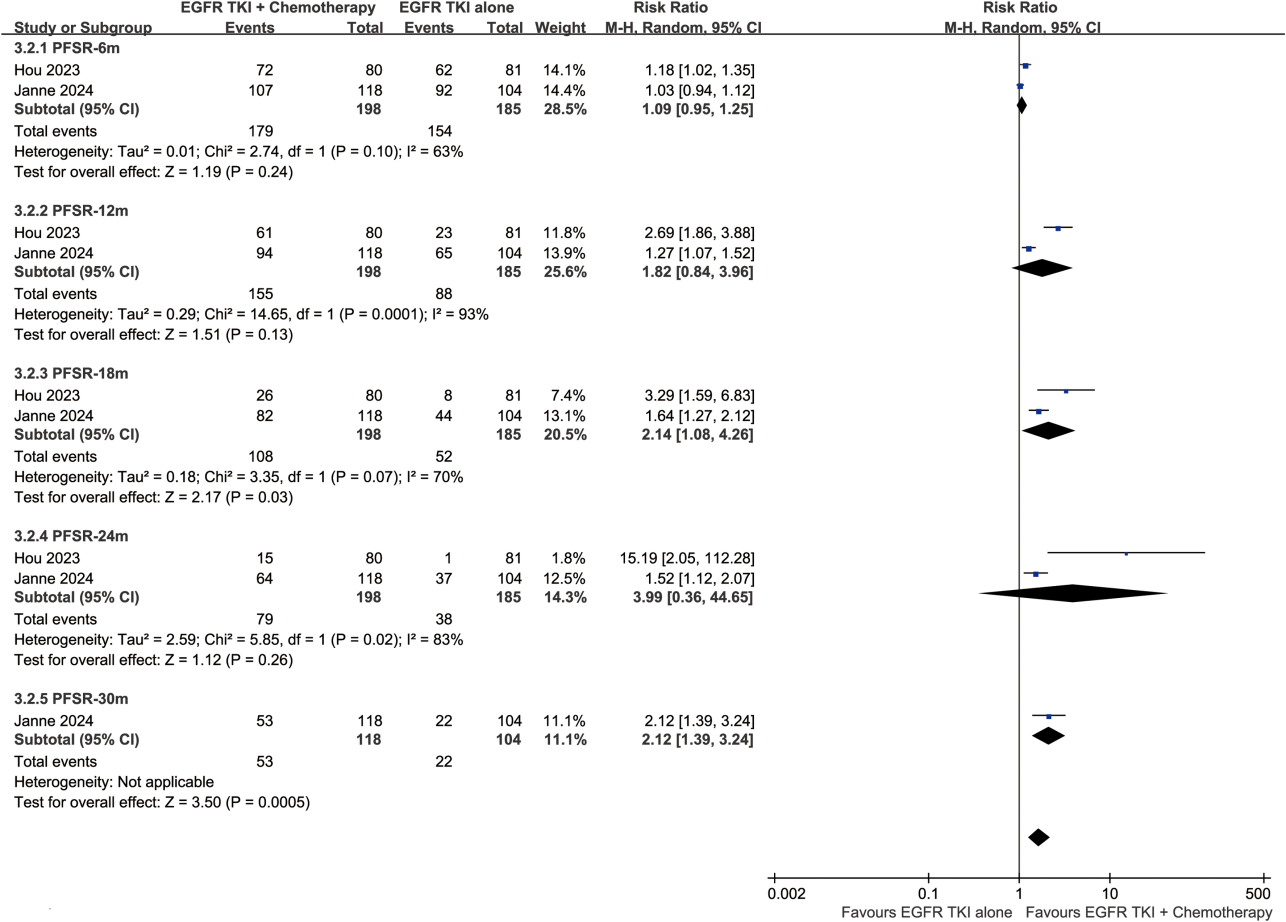
Forest plots of PFSR at 3-30 months associated with ETC versus ET.

The PFS was better in the ETC group (HR: 0.42 [0.34, 0.52]) (**Figure 4**). The progression-free survival rate (PFSR) also tended to favor the ETC group at 6 to 30 months (**Figure 5**).

The CNS-PFS was better in the ETC group (HR: 0.42 [0.31, 0.57]) (**Figure 4**). The central nervous system progression-free survival rate (CNS-PFSR) also tended to favor the ETC group at 6 to 30 months (**Supplementary Figure S2**).

### Subgroup analysis of survival

The survival advantages of OS, PFS, and CNS-PFS in the ETC group were confirmed in almost all subgroups according to age, sex, ECOG PS, EGFR mutation type, extracranial metastases, and EGFR TKIs. ECOG PS = 0, EGFR mutation - Ex19del, and a large intracranial tumor size < 20mm might be favorable factors for the ETC group (**Table 2**, **Supplementary Figures S3**–**S5**).

**Table 2:** Subgroup analysis of overall survival, progression-free survival, and CNS-progression-free survival.

Subgroups	Overall survival		Progression-free survival		CNS-Progression-free survival	
	HR (95% CI)	*P*	HR (95% CI)	*P*	HR (95% CI)	*P*
**All patients**	0.64 [0.48, 0.87]	0.004	0.42 [0.34, 0.52]	<0.00001	0.42 [0.31, 0.57]	<0.00001
Age						
< 65 years	0.64 [0.40, 1.02]	0.06	0.40 [0.26, 0.62]	<0.0001	0.40 [0.26, 0.61]	<0.0001
> 65 years	1.05 [0.40, 2.75]	0.92	0.42 [0.15, 1.19]	0.1	0.21 [0.06, 0.74]	0.01
Sex						
Female	0.64 [0.35, 1.17]	0.15	0.33 [0.18, 0.60]	0.0003	0.28 [0.16, 0.49]	<0.0001
Male	0.60 [0.34, 1.07]	0.08	0.45 [0.26, 0.78]	0.004	0.43 [0.25, 0.73]	0.002
Smoking status						
Smoker	0.77 [0.35, 1.69]	0.51	0.43 [0.20, 0.92]	0.03	0.49 [0.24, 1.00]	0.05
Non-smoker	0.56 [0.34, 0.94]	0.03	0.36 [0.22, 0.58]	<0.0001	0.28 [0.18, 0.45]	<0.00001
ECOG PS						
0	0.36 [0.13, 0.99]	0.05	0.31 [0.13, 0.73]	0.008	0.20 [0.09, 0.46]	0.0001
1	0.78 [0.49, 1.24]	0.29	0.42 [0.27, 0.66]	0.0001	0.43 [0.28, 0.66]	0.0001
Large intracranial tumor size						
< 20mm	0.53 [0.32, 0.88]	0.01	0.32 [0.18, 0.57]	0.0001	0.31 [0.19, 0.51]	<0.00001
> 20mm	0.97 [0.46, 2.04]	0.94	0.43 [0.24, 0.77]	0.005	0.44 [0.23, 0.86]	0.02
EGFR mutation						
Ex19del	0.40 [0.22, 0.73]	0.003	0.39 [0.24, 0.63]	0.0001	0.29 [0.17, 0.50]	<0.0001
L858R	0.83 [0.45, 1.54]	0.55	0.34 [0.15, 0.77]	0.009	0.34 [0.19, 0.62]	0.0004
Extracranial metastases						
Yes	0.54 [0.33, 0.88]	0.01	0.47 [0.33, 0.66]	<0.0001	0.35 [0.22, 0.55]	<0.00001
No	0.76 [0.31, 1.88]	0.55	0.39 [0.30, 0.52]	<0.00001	0.30 [0.14, 0.65]	0.002
EGFR TKIs						
Osimertinib	–	–	–	–	0.58 [0.33, 1.01]	0.06
Gefitinib	–	–	–	–	0.36 [0.25, 0.52]	<0.00001

CI, Confidence interval; CNS, Central Nervous System; ECOG, Eastern Cooperative Oncology Group; EGFR, Epidermal growth factor receptor; HR, Hazard ratio; P, Probability; TKIs, Tyrosine kinase inhibitors.

### Responses

In the analysis of overall responses, the overall response rate (ORR) (RR: 1.25 [1.02, 1.52]) and partial response (PR) (RR: 1.25 [1.02, 1.52]) were higher in the ETC group. The disease control rate (DCR) was similar between the two groups. The stable disease (SD) (RR: 0.49 [0.26, 0.90]) was higher in the ET group (**Supplementary Figure S6**).

In the analysis of CNS responses, the CNS-ORR (RR: 1.19 [0.93, 1.51]) and CNS-CR (RR: 1.31 [1.02, 1.70]) were higher in the ETC group. The CNS-DCR, CNS-PR, and CNS-SD were similar between the two groups (**Supplementary Figure S7**).

### Progression status

**Figure 6:**
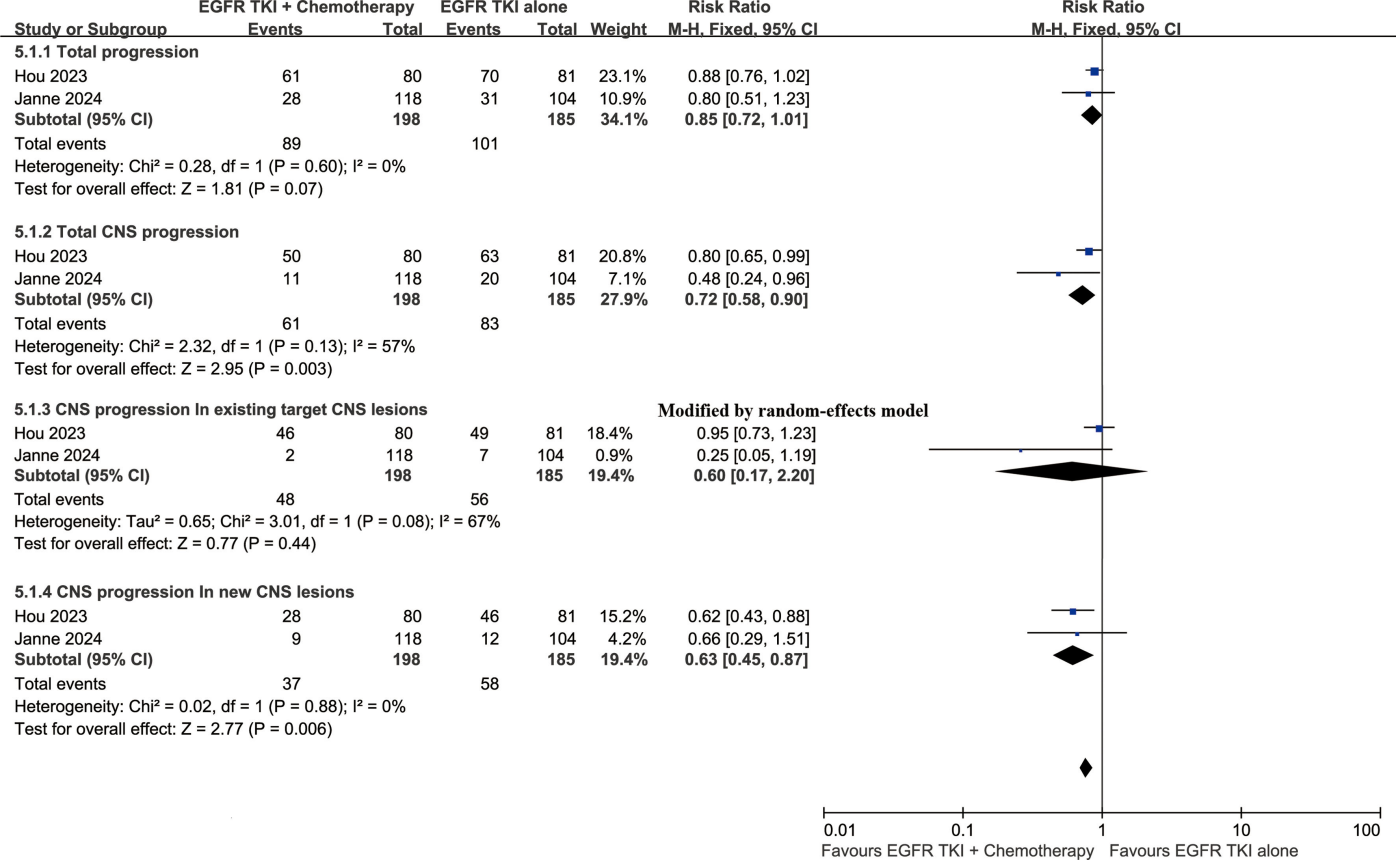
Forest plots of progression status associated with ETC versus ET.

At the cutoff time of the studies, the total progression (RR: 0.85 [0.72, 1.01]) and CNS progression (RR: 0.72 [0.58, 0.90]) tended to favor the ETC group. The addition of chemotherapy was particularly effective in controlling newly developed intracranial lesions (RR: 0.63 [0.45, 0.87]) (**Figure 6**).

### Safety

The rates of grade 3-5 AEs (RR: 2.10 [1.59, 2.77]), serious AEs (RR: 1.69 [1.10, 2.59]), discontinuation due to AEs (RR: 7.73 [3.57, 16.77]), and grade 3-5 treatment-related AEs (TRAEs) (RR: 3.65 [2.17, 6.15]) were higher in the ETC group. The total AEs, fatal AEs, dose interruption due to AEs, total TRAEs, serious TRAEs, and fatal TRAEs tended to favor the ET group without statistical differences (**Table 3**, **Supplementary Figure S8**).

**Table 3:** Summary of adverse events.

Adverse events	ETC		ET		Risk ratio [95% CI]	P
	Event/total	%	Event/total	%		
Total adverse events	196/198	98.99%	177/185	95.68%	1.04 [0.96, 1.12]	0.39
Grade 3-5 adverse events	107/198	54.04%	47/185	25.41%	2.10 [1.59, 2.77]	<0.00001
Serious adverse events	44/118	37.29%	23/104	22.12%	1.69 [1.10, 2.59]	0.02
Fatal adverse events	7/118	5.93%	3/104	2.88%	2.06 [0.55, 7.75]	0.29
Discontinuation due to adverse events	56/198	28.28%	6/185	3.24%	7.73 [3.57, 16.77]	<0.00001
Dose interruption due to adverse events	10/80	12.50%	7/81	8.64%	1.45 [0.58, 3.61]	0.43
Treatment-related adverse events	112/118	94.92%	92/104	88.46%	1.07 [0.99, 1.16]	0.09
Grade 3-5 treatment-related adverse events	58/118	49.15%	14/104	13.46%	3.65 [2.17, 6.15]	<0.00001
Serious treatment-related adverse events	20/118	16.95%	9/104	8.65%	1.96 [0.93, 4.11]	0.08
Fatal treatment-related adverse events	3/198	1.52%	0/185	0.00%	3.75 [0.42, 33.49]	0.24

CI, Confidence interval; ET, EGFR tyrosine kinase inhibitors alone; ETC, EGFR tyrosine kinase inhibitors in combination of chemotherapy; P, Probability.

In the analysis of any grade AEs, more cases of anorexia, alanine aminotransferase increase, neutropenia, alkaline phosphatase increase, nausea, fatigue, vomiting, blood creatinine increase, thrombocytopenia, and constipation were found in the ETC group (**Table 4**, **Supplementary Figure S9**).

**Table 4:** Any grade adverse events.

Adverse events	ETC		ET		Risk ratio [95% CI]	P
	Event/total	%	Event/total	%		
Anorexia	58/80	72.50%	15/81	18.52%	3.92 [2.43, 6.30]	<0.00001
Alanine aminotransferase increase	56/80	70.00%	42/81	51.85%	1.35 [1.05, 1.74]	0.02
Leukopenia	50/80	62.50%	6/81	7.41%	8.44 [3.84, 18.56]	<0.00001
Neutropenia	49/80	61.25%	6/81	7.41%	8.27 [3.75, 18.21]	<0.00001
Aspartate aminotransferase increase	46/80	57.50%	41/81	50.62%	1.14 [0.85, 1.51]	0.38
Anemia	45/80	56.25%	25/81	30.86%	1.82 [1.25, 2.66]	0.002
Alkaline phosphatase increase	45/80	56.25%	31/81	38.27%	1.47 [1.05, 2.06]	0.03
Rash	45/80	56.25%	45/81	55.56%	1.01 [0.77, 1.33]	0.93
Nausea	40/80	50.00%	3/81	3.70%	13.50 [4.35, 41.87]	<0.00001
Fatigue	37/80	46.25%	20/81	24.69%	1.87 [1.20, 2.93]	0.006
Vomiting	32/80	40.00%	1/81	1.23%	32.40 [4.54, 231.46]	0.0005
Hypoalbuminemia	30/80	37.50%	21/81	25.93%	1.45 [0.91, 2.30]	0.12
Pruritus	26/80	32.50%	29/81	35.80%	0.91 [0.59, 1.40]	0.66
Blood creatinine increase	22/80	27.50%	6/81	7.41%	3.71 [1.59, 8.67]	0.002
Diarrhea	20/80	25.00%	26/81	32.10%	0.78 [0.48, 1.28]	0.32
Thrombocytopenia	19/80	23.75%	2/81	2.47%	9.62 [2.32, 39.95]	0.002
Hypocalcemia	19/80	23.75%	13/81	16.05%	1.48 [0.78, 2.79]	0.23
Constipation	18/80	22.50%	4/81	4.94%	4.56 [1.61, 12.87]	0.004
Hypokalemia	11/80	13.75%	16/81	19.75%	0.70 [0.34, 1.41]	0.31
Hyponatremia	9/80	11.25%	13/81	16.05%	0.70 [0.32, 1.55]	0.38
Blood bilirubin increase	7/80	8.75%	11/81	13.58%	0.64 [0.26, 1.58]	0.34
Paronychia	6/80	7.50%	9/81	11.11%	0.68 [0.25, 1.81]	0.43
Hypercalcemia	5/80	6.25%	1/81	1.23%	5.06 [0.60, 42.37]	0.13
Hyperkalemia	3/80	3.75%	1/81	1.23%	3.04 [0.32, 28.59]	0.33

CI, Confidence interval; ET, EGFR tyrosine kinase inhibitors alone; ETC, EGFR tyrosine kinase inhibitors in combination of chemotherapy; P, Probability.

In the analysis of grade 3-5 AEs, most AEs tended to favor the ET group without statistical differences. The top 5 grade 3-5 AEs in the ETC group were alanine aminotransferase increase (11.25%), neutropenia (7.5%), nausea (7.5%), anorexia (5%), and diarrhea (5%) (**Supplementary Table S5**, **Supplementary Figure S10**).

### Sensitivity analysis

Sensitivity analyses of OS and PFS were performed, demonstrating that excluding any single study had no impact on the credibility of the results (**Supplementary Figure S11**).

### Publication bias

**Figure 7:**
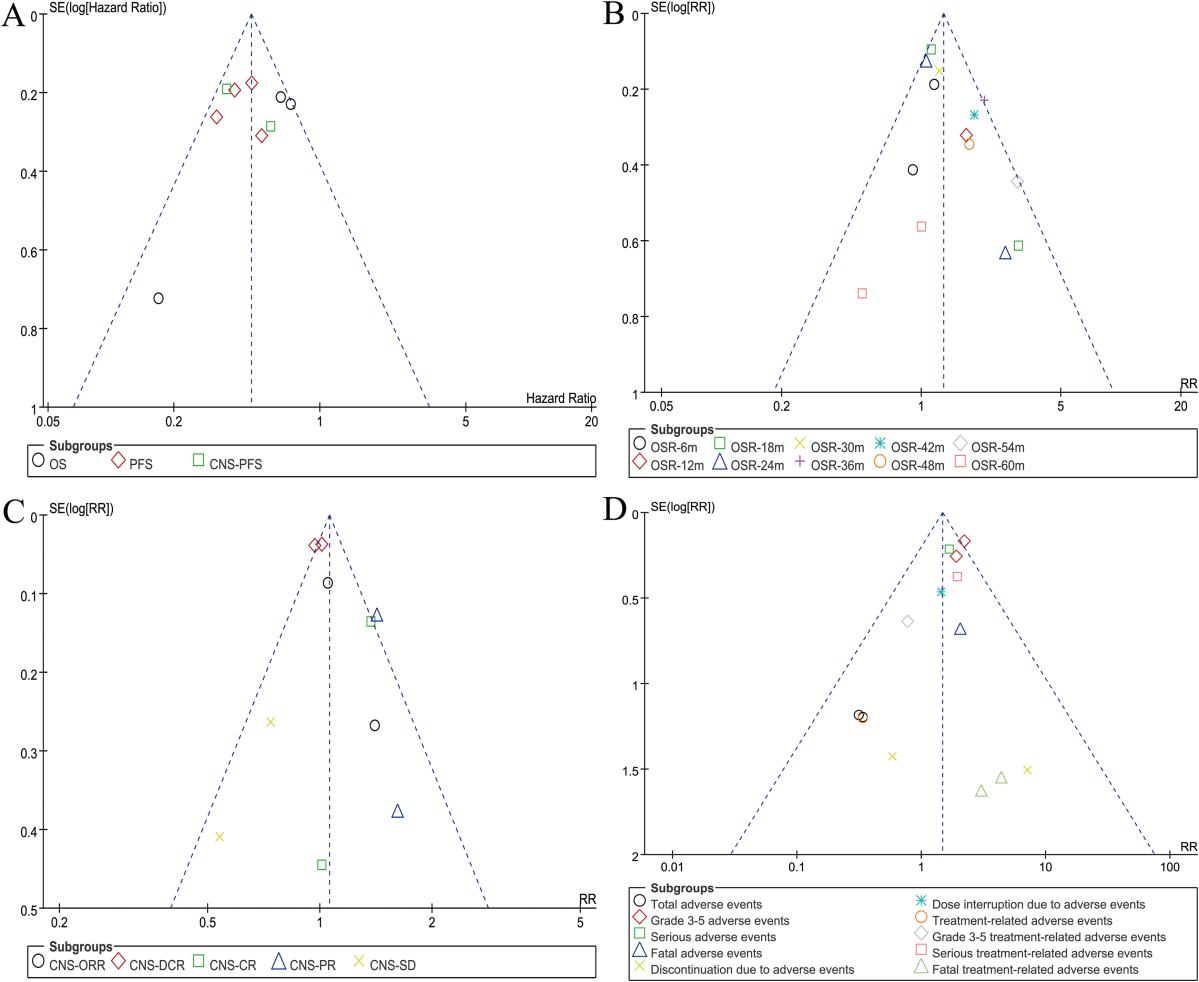
Funnel plots of overall survival **(A)**, overall survival rate **(B)**, CNS responses **(C)**, and safety summary **(D)**.

Funnel plots of survival, OSR, CNS responses, and safety summary were constructed. It was observed that studies were evenly distributed on both sides of the funnel plot, with almost all falling within its confines. This suggested minimal publication bias in this study (**Figure 7**). Egger’s and Begg’s tests based on OS and PFS also showed no significant publication bias (**Supplementary Figure S11**).

## Discussion

In recent years, for advanced NSCLC patients with EGFR mutations, EGFR-TKI has become the standard first-line treatment, replacing chemotherapy. The antitumor mechanisms of EGFR-TKI and chemotherapy differ, and relevant preclinical and clinical studies have confirmed the potential of combination therapy (21, 22). Numerous studies have demonstrated that combination therapy can achieve better OS and PFS for advanced EGFR-positive NSCLC (23, 24). The survival advantage of combination therapy has also been confirmed by numerous meta-analyses, not only compared to chemotherapy, but also compared to EGFR-TKI monotherapy (**Supplementary Table S6**). However, whether this conclusion applies to patients with BM remains controversial in clinical practice. This meta-analysis, for the first time, compared the ETC and ET treatments in EGFR-positive NSCLC patients with BM based on RCTs. The results showed that the ETC group exhibited better survival, which was confirmed across almost all subgroups. Additionally, the overall objective response rate (ORR) and CNS-ORR tended to favor the ETC group. However, the addition of chemotherapy also led to more grade 3-5/serious AEs.

The greatest advantage of ETC over the ET group lies in its superior survival outcomes (OS, PFS, and CNS-PFS). This conclusion was supported by evidence from studies by Lou et al. and Hou et al. (7, 8). Preclinical studies had found that the ETC exerted a synergistic inhibitory effect on EGFR-sensitive cells (25, 26), as confirmed in trials such as CALGB30406, FASTACT-2, and NEJ005/TCOG0902 (27–29). NEJ005 also indicated a significant advantage in OS for EGFR-TKI combined with chemotherapy compared to sequential treatment, although the difference in PFS between patients was not significant (29). The enhanced efficacy of ETC might be related to the reduction of EGFR T790M mutation, which could promote resistance to EGFR TKIs (30). Another reason was the better drug response observed in the ETC group. Our study indicated that the ETC group exhibits superior ORR and CNS-ORR. The survival advantages of OS, PFS, and CNS-PFS in the ETC group were confirmed across almost all subgroups, particularly in patients with ECOG performance status = 0, EGFR mutation - Ex19del, and large intracranial tumor size < 20mm. In conclusion, due to its superior systemic and intracranial efficacy, we believed that combination therapy should be considered as the preferred treatment for EGFR-positive NSCLC patients with BM.

The main concern among clinical physicians regarding the ETC regimen is the potential for chemotherapy to induce more severe AEs (31, 32). Our study indicated that the rates of grade 3-5 AEs, serious AEs, discontinuation due to AEs, and grade 3-5 TRAEs were higher in the ETC group. The top 5 grade 3-5 AEs in the ETC group were alanine aminotransferase increase (11.25%), neutropenia (7.5%), nausea (7.5%), anorexia (5%), and diarrhea (5%). Studies by Janne et al. and Hou et al. had also found a significant increase in AEs occurrence in the ETC group, primarily concentrated in any grade AEs (8, 9). Although most grade 3-5 AEs tended to favor the ET group, they did not reach statistical significance. Therefore, we believe that the combined use of EGFR TKIs and chemotherapy, while potentially increasing the occurrence of AEs, remains within an acceptable range in terms of incidence and severity.

Limitations of this meta-analysis include: 1. The inclusion criteria were limited to English-published studies, potentially reducing the comprehensiveness of the analysis. 2. Some survival data were collected from subgroup analyses of large-scale RCTs, where differences in baseline characteristics among patients might affect the reliability of the data. 3. The number of studies included in some result analyses was small, compromising the clinical guidance value of the final results. 4. The majority of studies included in the analysis were conducted in Asia, potentially limiting the applicability of the data analysis conclusions to patients in other regions. 5. The included studies used different evaluation criteria to assess AEs, which would increase the heterogeneity of AEs analysis.

## Conclusion

ETC appears to outperform ET in treating EGFR-positive NSCLC patients with BM, showing improvements in OS, PFS, CNS-PFS, and responses. The survival advantages of OS, PFS, and CNS-PFS in the ETC group were observed across nearly all subgroups, particularly in those with ECOG performance status = 0, EGFR mutation - Ex19del, and large intracranial tumor size < 20mm. However, the poorer safety profile of ETC should also be considered. Given the aforementioned limitations, it is essential to conduct additional high-quality randomized controlled trials to validate these conclusions.

## Data Availability

The original contributions presented in the study are included in the article/**Supplementary Material**. Further inquiries can be directed to the corresponding author.

## Supplementary material

Supplementary Figure 1 Cochrane Risk Assessment.

Supplementary Figure 2 Forest plots of CNS-PFSR at 3-30 months associated with ETC versus ET.

Supplementary Figure 3 Forest plots of subgroup analysis of overall survival associated with ETC versus ET.

Supplementary Figure 4 Forest plots of subgroup analysis of progression-free survival associated with ETC versus ET.

Supplementary Figure 5 Forest plots of subgroup analysis of CNS-progression-free survival associated with ETC versus ET.

Supplementary Figure 6 Forest plots of overall responses associated with ETC versus ET.

Supplementary Figure 7 Forest plots of CNS responses associated with ETC versus ET.

Supplementary Figure 8 Forest plots of safety summary associated with ETC versus ET.

Supplementary Figure 9 Forest plots of any grade adverse events associated with ETC versus ET.

Supplementary Figure 10 Forest plots of grade 3-5 adverse events associated with ETC versus ET.

Supplementary Figure 11 Sensitivity analysis of overall survival **(A)** and progression-free survival **(B)**.

Supplementary Figure 12 Egger’s and Begg’s tests based on OS **(A)** and PFS **(B)**.
