# Supplementary figures and images for: The benefit and risk of addition of chemotherapy to EGFR tyrosine kinase inhibitors for EGFR-positive non-small cell lung cancer patients with brain metastases: a meta-analysis based on randomized controlled trials
Source: Front Oncol. 2024 Oct 21;14:1448336. doi: 10.3389/fonc.2024.1448336 (PMC11532100; doi:10.3389/fonc.2024.1448336)

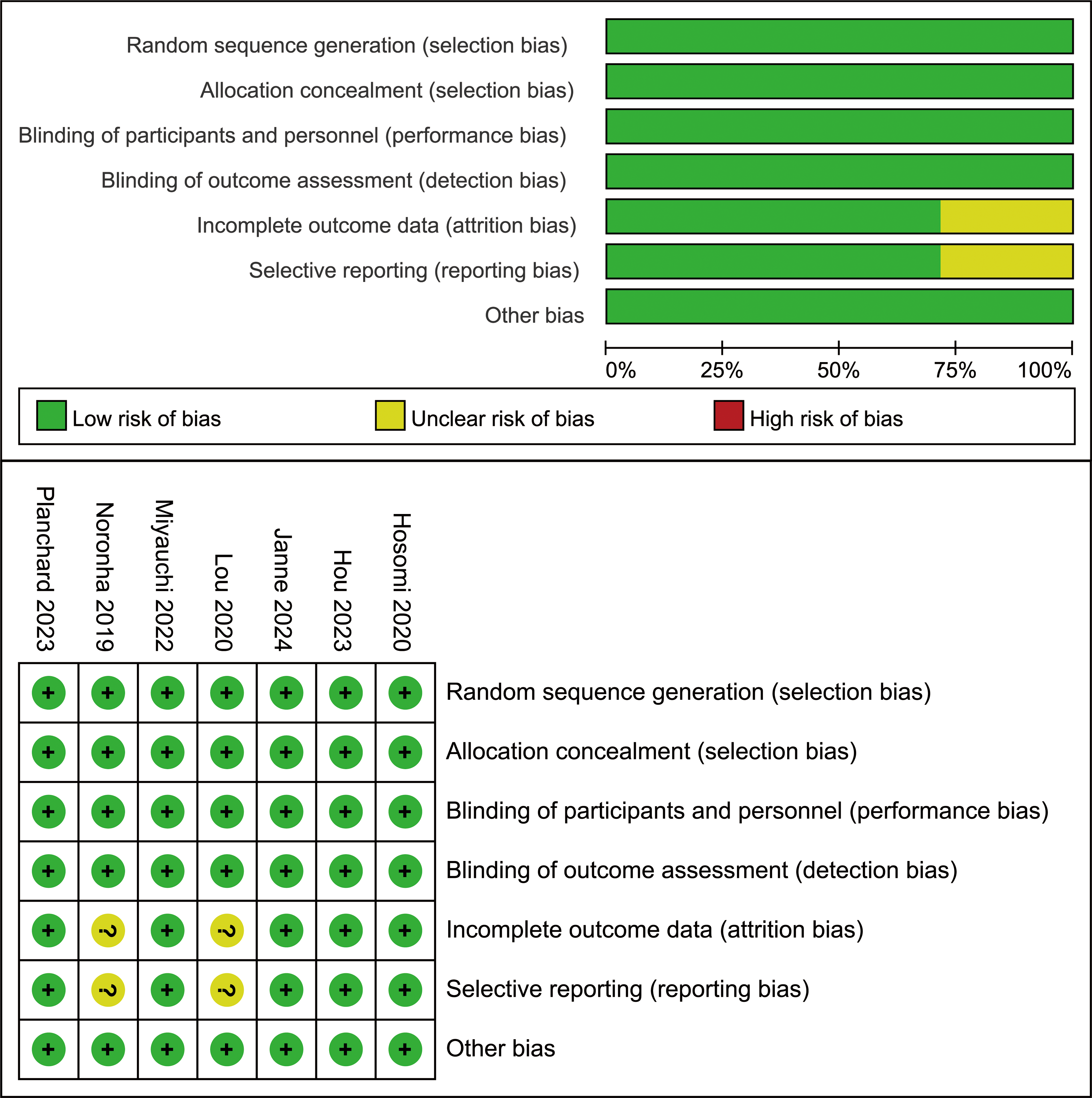

Supplement: Supplementary Figure 1 — Cochrane Risk Assessment. [file Image1.tif]

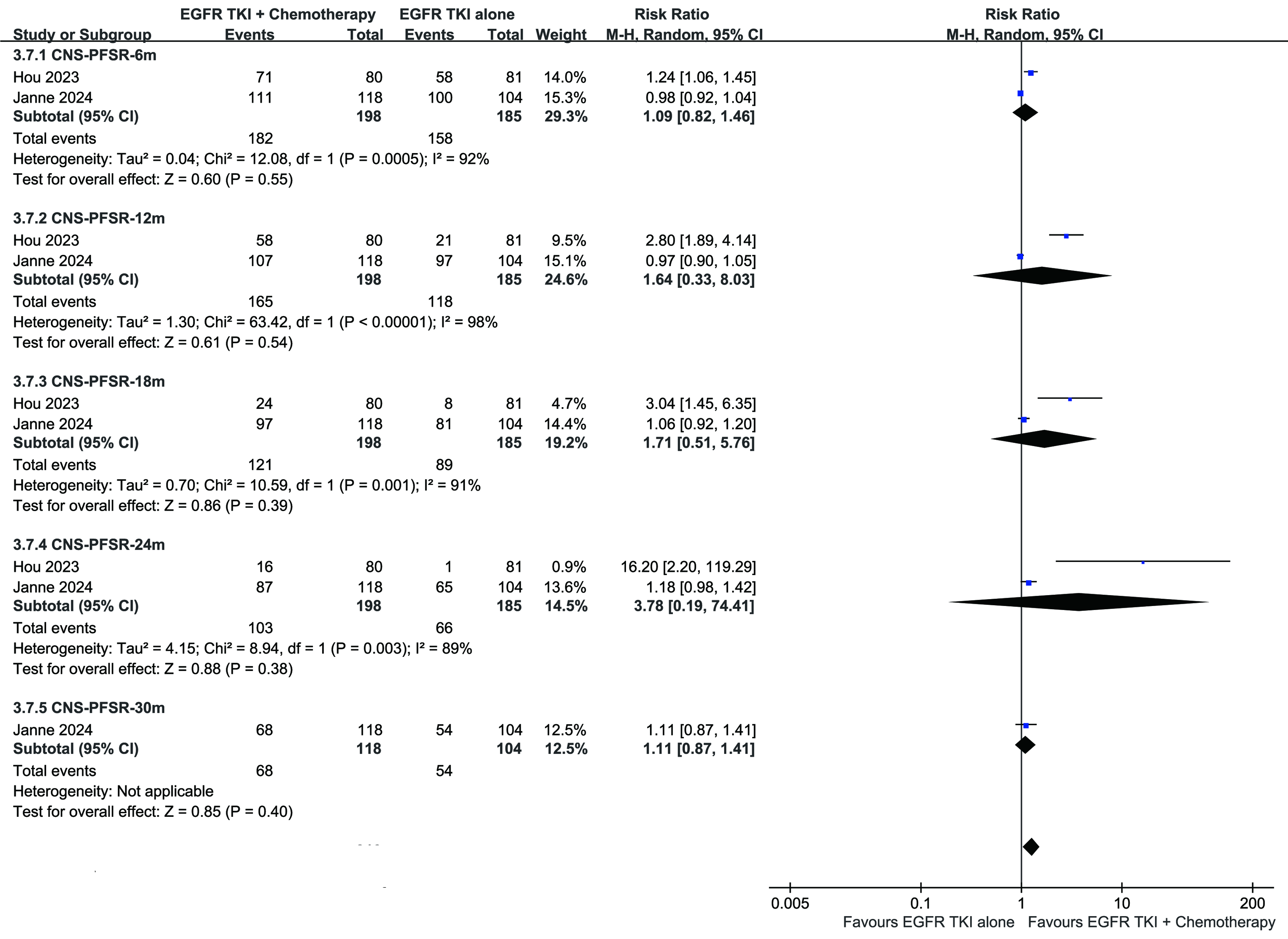

Supplement: Supplementary Figure 2 — Forest plots of CNS-PFSR at 3-30 months associated with ETC versus ET. [file Image2.tif]

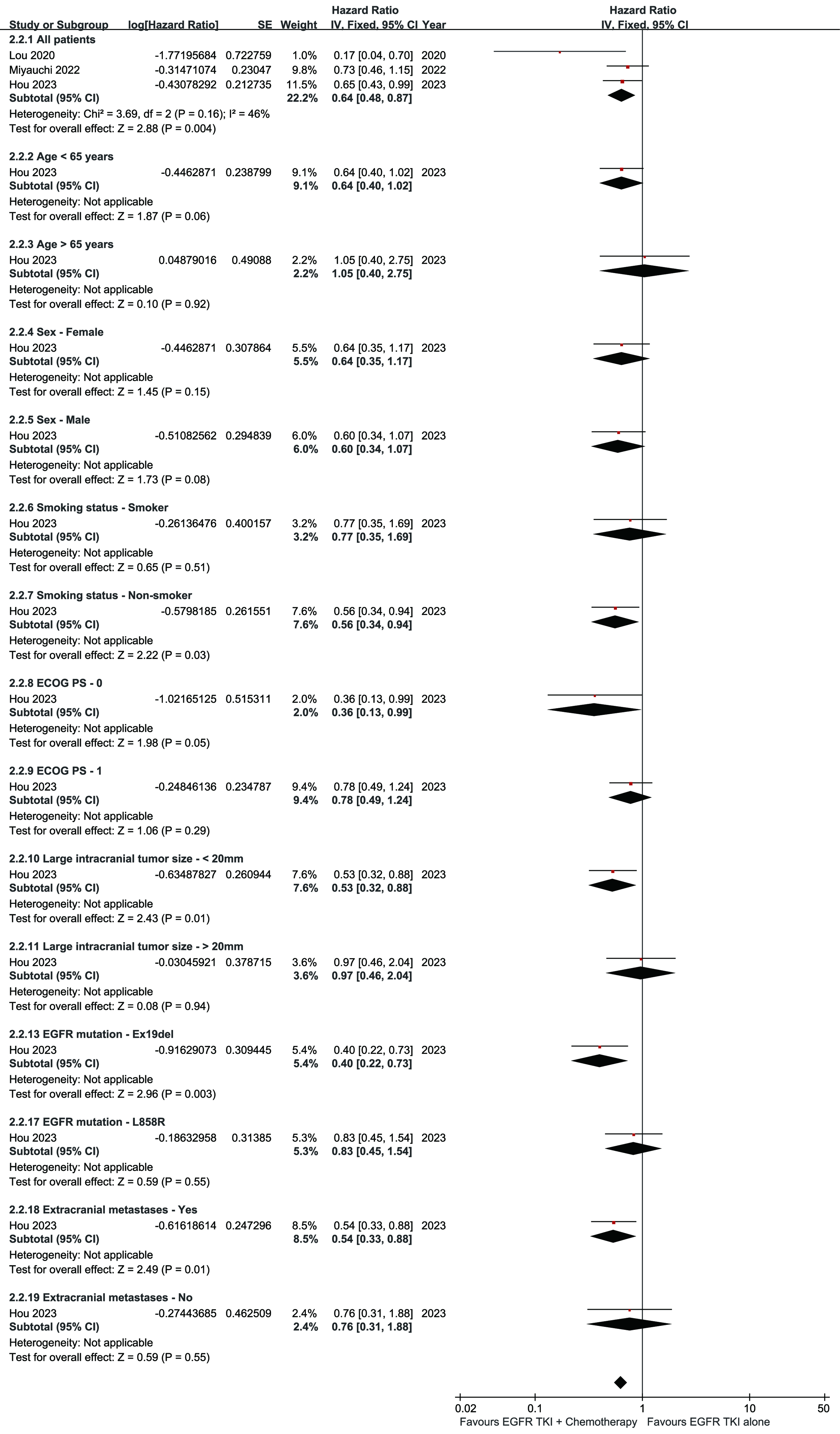

Supplement: Supplementary Figure 3 — Forest plots of subgroup analysis of overall survival associated with ETC versus ET. [file Image3.tif]

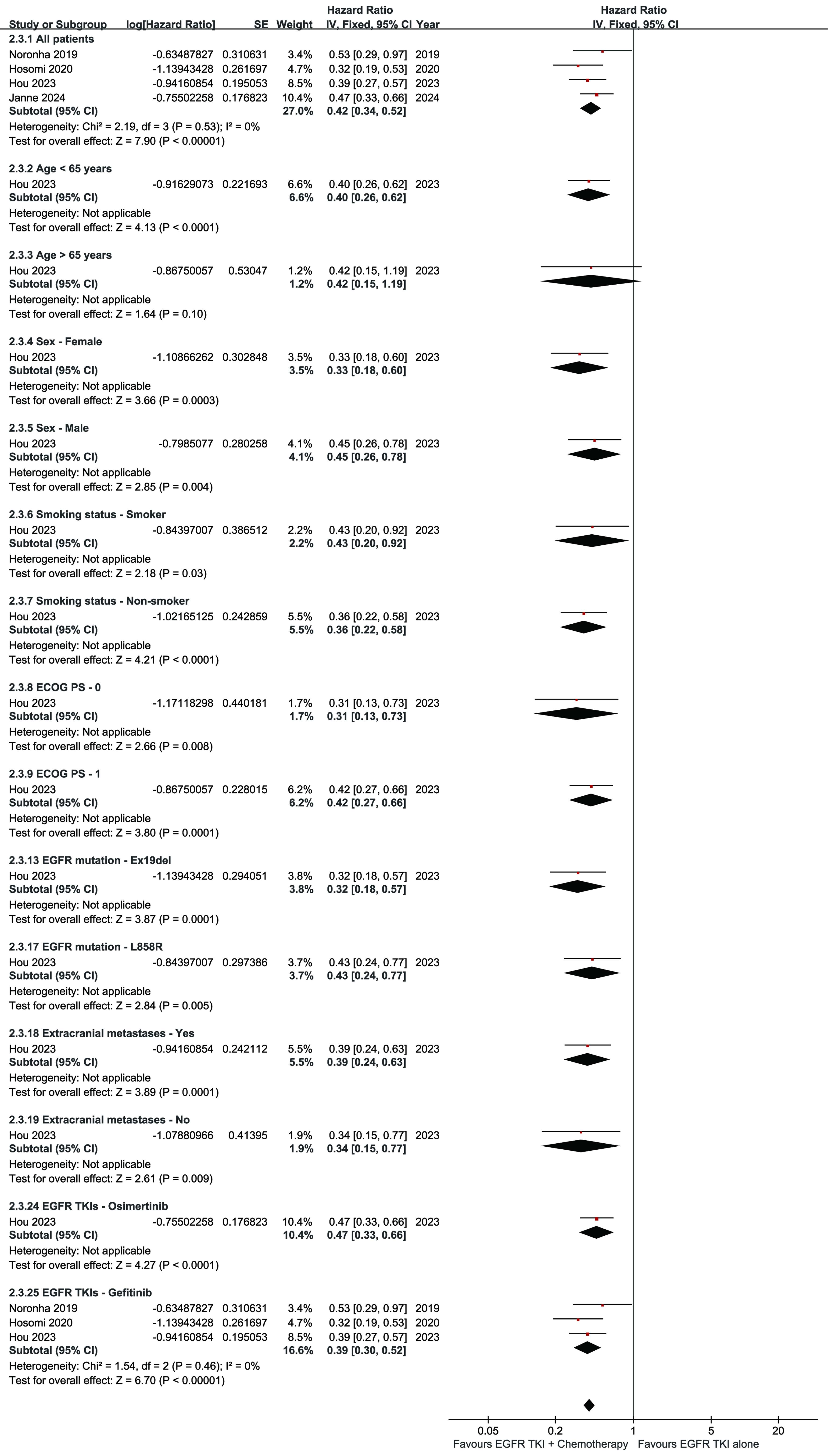

Supplement: Supplementary Figure 4 — Forest plots of subgroup analysis of progression-free survival associated with ETC versus ET. [file Image4.tif]

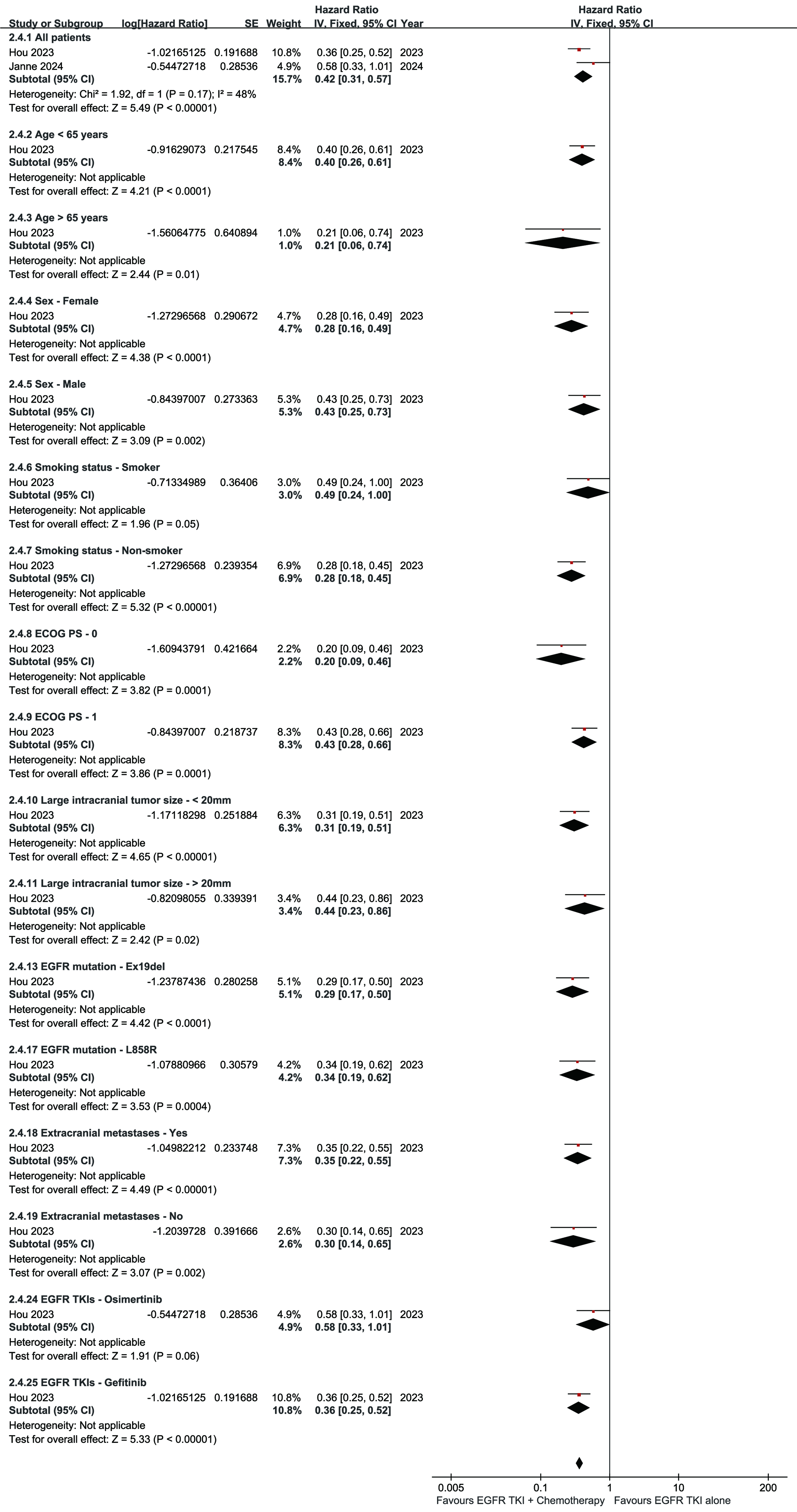

Supplement: Supplementary Figure 5 — Forest plots of subgroup analysis of CNS-progression-free survival associated with ETC versus ET. [file Image5.tif]

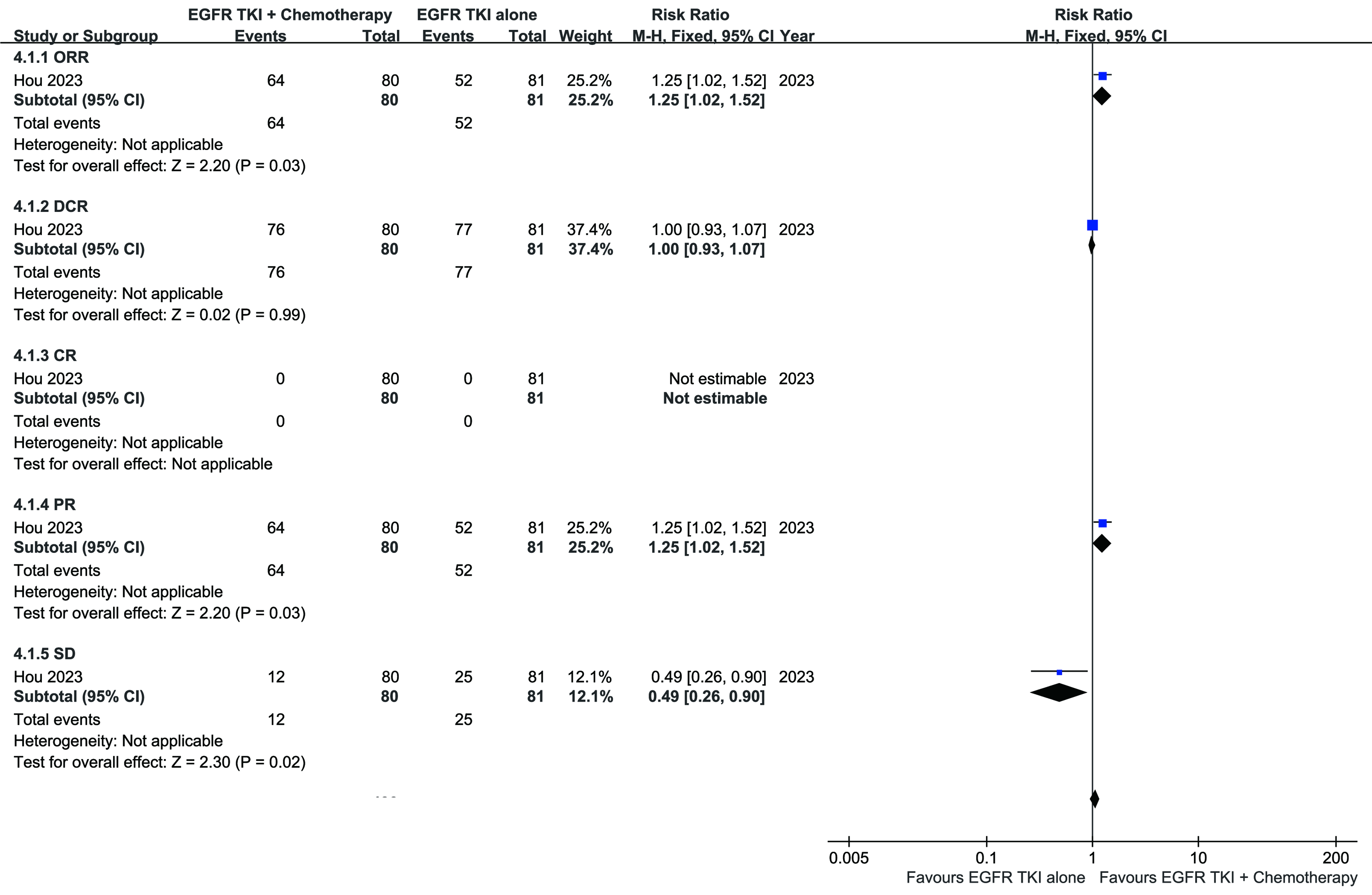

Supplement: Supplementary Figure 6 — Forest plots of overall responses associated with ETC versus ET. [file Image6.tif]

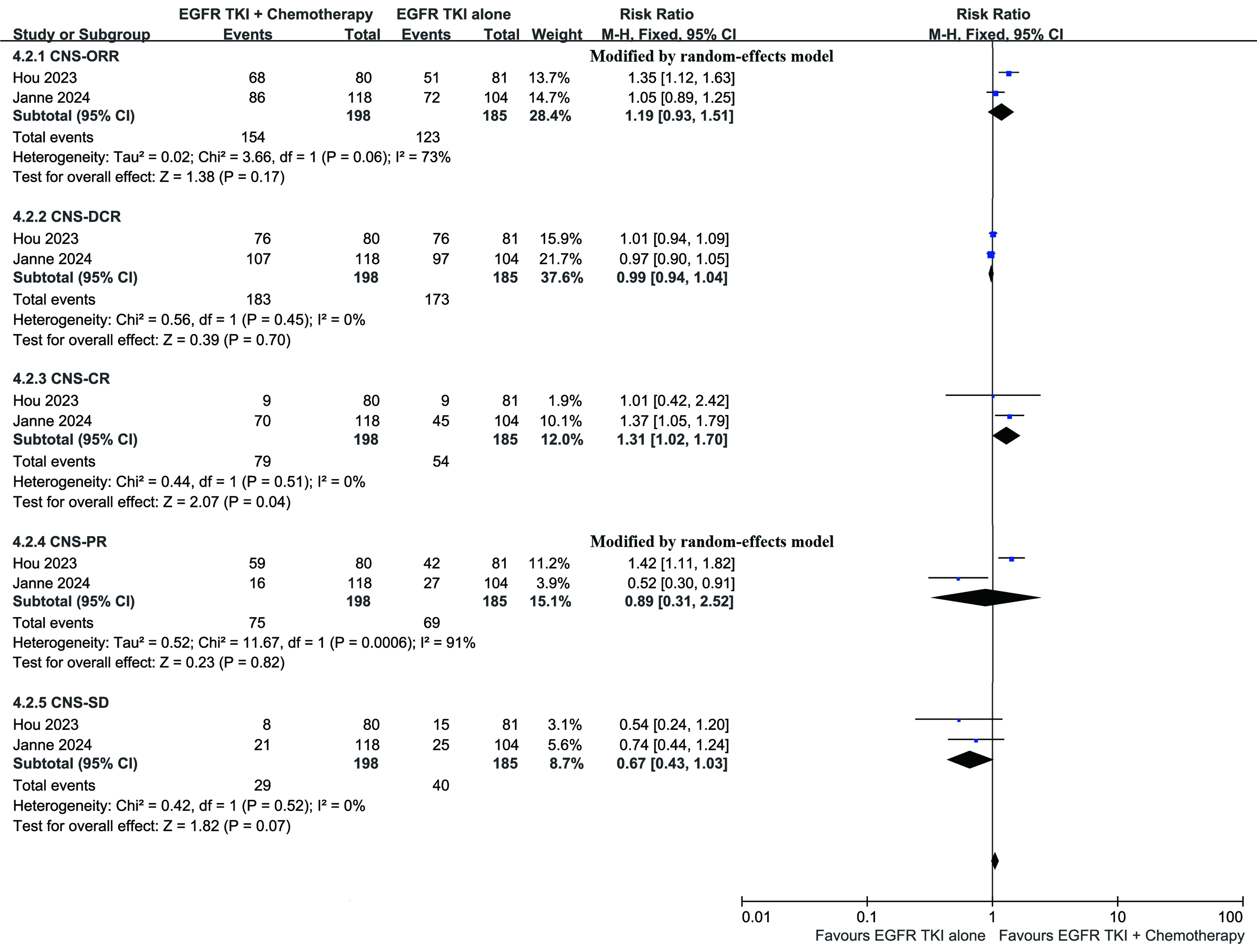

Supplement: Supplementary Figure 7 — Forest plots of CNS responses associated with ETC versus ET. [file Image7.tif]

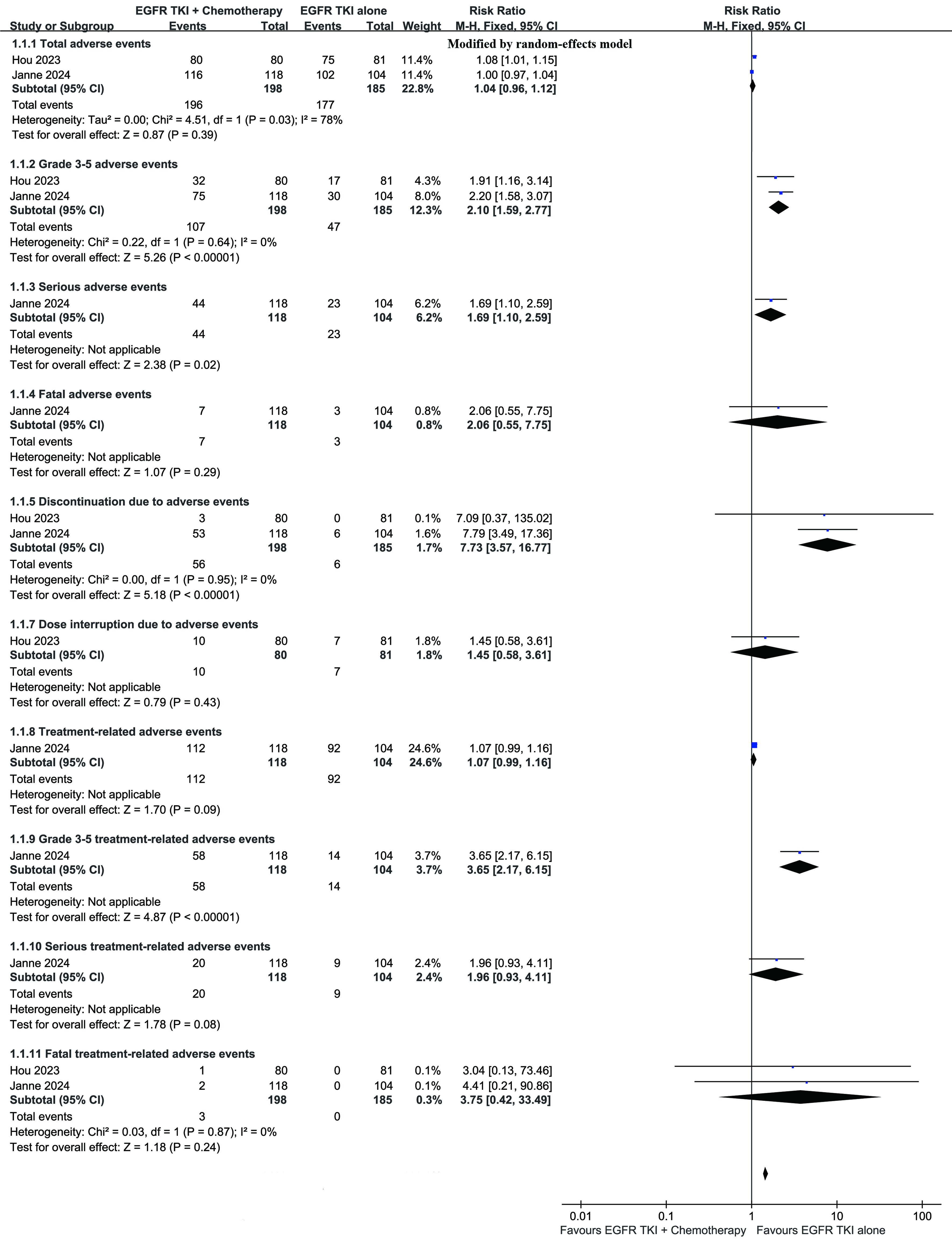

Supplement: Supplementary Figure 8 — Forest plots of safety summary associated with ETC versus ET. [file Image8.tif]

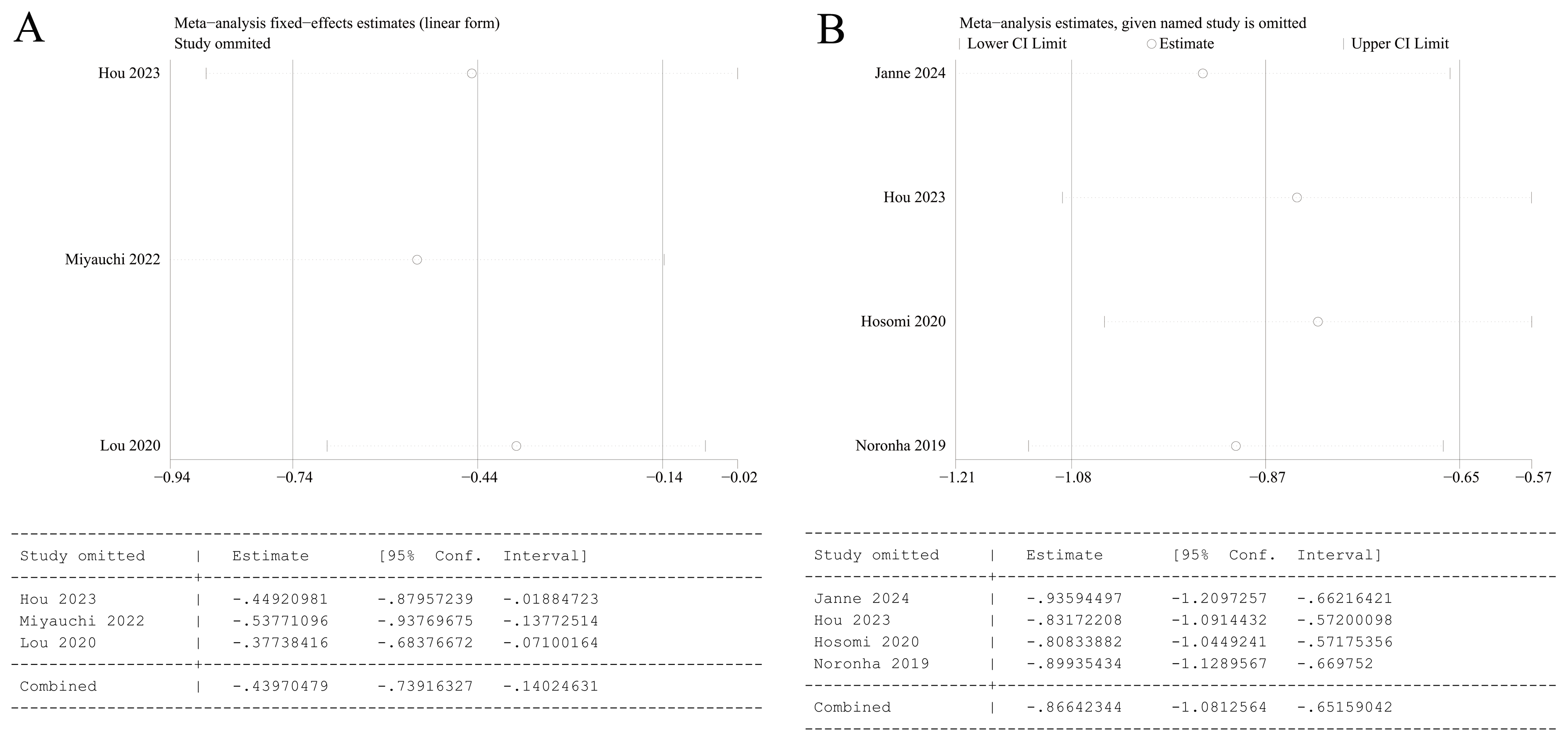

Supplement: Supplementary Figure 11 — Sensitivity analysis of overall survival (A) and progression-free survival (B). [file Image11.tif]

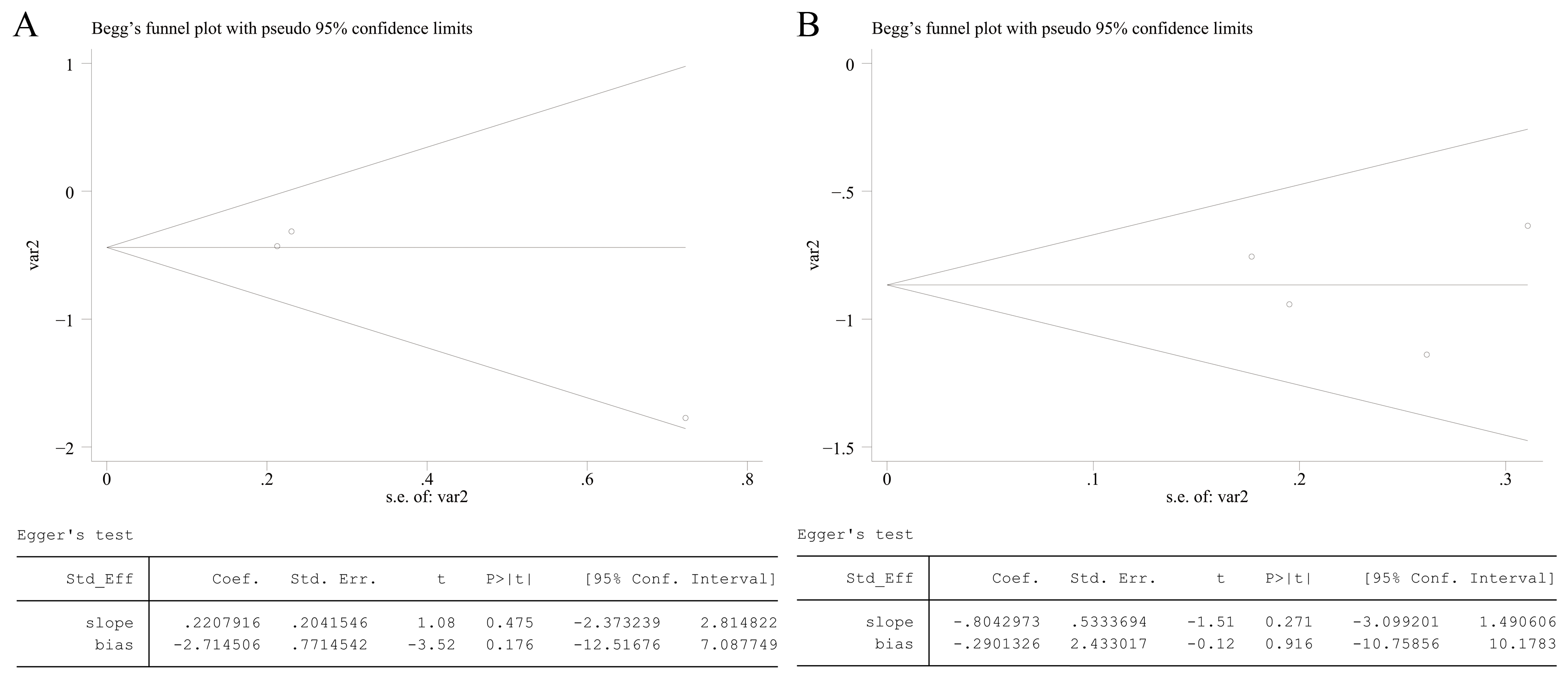

Supplement: Supplementary Figure 12 — Egger’s and Begg’s tests based on OS (A) and PFS (B). [file Image12.tif]
