# Supplementary material for: The benefit and risk of addition of chemotherapy to EGFR tyrosine kinase inhibitors for EGFR-positive non-small cell lung cancer patients with brain metastases: a meta-analysis based on randomized controlled trials
Source: Front Oncol. 2024 Oct 21;14:1448336. doi: 10.3389/fonc.2024.1448336 (PMC11532100; doi:10.3389/fonc.2024.1448336)
Supplement: Supplementary file 13 [file Table1.docx]

**Table S1** Search strategy.

| **PubMed**  The database was searched on August 27, 2024, n=940.  Search Strategy:  #1 EGFR[Title/Abstract] OR Epidermal growth factor receptor[Title/Abstract] Sort by: Most Recent n = 112,353  #2 Chemotherapy[Title/Abstract] OR Cisplatin[Title/Abstract] OR Carboplatin[Title/Abstract] OR Pemetrexed[Title/Abstract] OR Gemcitabine[Title/Abstract] OR Paclitaxel[Title/Abstract] OR Docetaxel[Title/Abstract] OR Vinorelbine[Title/Abstract] OR Irinotecan[Title/Abstract] OR Topotecan[Title/Abstract] OR Etoposide[Title/Abstract] OR Doxorubicin[Title/Abstract] Sort by: Most Recent n = 592,571  #3 Lung cancer[Title/Abstract] OR NSCLC[Title/Abstract] OR Lung adenocarcinoma[Title/Abstract] OR Lung squamous cell carcinoma[Title/Abstract] Sort by: Most Recent n = 238,007  #4 Randomized[Title/Abstract] OR Randomly[Title/Abstract] OR Randomised[Title/Abstract] Sort by: Most Recent n = 1,192,486  #1 and #2 and #3 and #4 n = 940 |
| --- |
| **Web of Science**  The database was searched on August 27, 2024, 2024, n=3457.  Search Strategy:  (EGFR(Abstract) OR Epidermal growth factor receptor(Abstract)) AND (**Chemotherapy(Abstract) OR Cisplatin(Abstract) OR Carboplatin(Abstract) OR Pemetrexed(Abstract) OR Gemcitabine(Abstract) OR Paclitaxel(Abstract) OR Docetaxel(Abstract) OR Vinorelbine(Abstract) OR Irinotecan(Abstract) OR Topotecan(Abstract) OR Etoposide(Abstract) OR Doxorubicin(Abstract)**) AND **(**Lung cancer(Abstract) **OR** NSCLC(Abstract) **OR**  Lung adenocarcinoma(Abstract**)) OR**  Lung squamous cell carcinoma(Abstract**)**) AND **(Randomly**(Abstract) **OR Randomised**(Abstract**) OR**  **Randomized** (Abstract**))** |
| **EMBASE**  The database was searched on August 27, 2024, 2024, n=1035.  Search Strategy:  (EGFR:ti,ab,kw OR Epidermal growth factor receptor:ti,ab,kw) AND (**Chemotherapy:ti,ab,kw OR Cisplatin:ti,ab,kw OR Carboplatin:ti,ab,kw OR Pemetrexed:ti,ab,kw OR Gemcitabine:ti,ab,kw OR Paclitaxel:ti,ab,kw OR Docetaxel:ti,ab,kw OR Vinorelbine:ti,ab,kw OR Irinotecan:ti,ab,kw OR Topotecan:ti,ab,kw OR Etoposide:ti,ab,kw OR Doxorubicin:ti,ab,kw**) AND **(**Lung cancer:ti,ab,kw **OR** NSCLC:ti,ab,kw **OR** Lung adenocarcinoma:ti,ab,kw**) OR** Lung squamous cell carcinoma:ti,ab,kw) AND **(Randomly**:ti,ab,kw **OR Randomised**:ti,ab,kw **OR**  **Randomized** :ti,ab,kw**)** |
| **Cochrane Library**  The database was searched on August 27, 2024, 2024, n=76.  Search Strategy:  (EGFR OR Epidermal growth factor receptor**)** in Title Abstract Keyword AND (Chemotherapy OR Cisplatin OR Carboplatin OR Pemetrexed OR Gemcitabine OR Paclitaxel OR Docetaxel OR Vinorelbine OR Irinotecan OR Topotecan OR Etoposide OR Doxorubicin**)** in Title Abstract Keyword AND (Lung cancer OR NSCLC OR Lung adenocarcinoma OR Lung squamous cell carcinoma**)** in Title Abstract Keyword AND (**Randomized OR Randomly OR Randomised)** in Title Abstract Keyword - (Word variations have been searched) |
| **ScienceDirect**  The database was searched on August 27, 2024, n=1877.  Search Strategy:  Title, abstract, keywords: ((“EGFR” OR “Epidermal growth factor receptor”) AND (“Chemotherapy” OR “Cisplatin” OR “Carboplatin” OR “Pemetrexed” OR “Gemcitabine” OR “Paclitaxel” OR “Docetaxel” OR “Vinorelbine” OR “Irinotecan” OR “Topotecan” OR “Etoposide” OR “Doxorubicin”) AND (“Lung cancer” OR “NSCLC” OR “Lung adenocarcinoma” OR “Lung squamous cell carcinoma”) AND (“**Randomized**” **OR Randomly**” **OR** “**Randomised**”)) |
| **Scopus**  The database was searched on August 27, 2024, n=369.  Search Strategy:  (TITLE-ABS-KEY (EGFR OR Epidermal growth factor receptor) AND TITLE-ABS-KEY (Chemotherapy OR Cisplatin OR Carboplatin OR Pemetrexed OR Gemcitabine OR Paclitaxel OR Docetaxel OR Vinorelbine OR Irinotecan OR Topotecan OR Etoposide OR Doxorubicin) AND TITLE-ABS-KEY (Lung cancer OR NSCLC OR Lung adenocarcinoma OR Lung squamous cell carcinoma) AND TITLE-ABS-KEY (**Randomized OR Randomly OR Randomised**)) |

**Note:** The combined text and medical subject heading (MeSH) terms used were: “EGFR”, “Chemotherapy”, “Lung cancer”, and “**Randomized**”.
