# Supplementary material for: The benefit and risk of addition of chemotherapy to EGFR tyrosine kinase inhibitors for EGFR-positive non-small cell lung cancer patients with brain metastases: a meta-analysis based on randomized controlled trials
Source: Front Oncol. 2024 Oct 21;14:1448336. doi: 10.3389/fonc.2024.1448336 (PMC11532100; doi:10.3389/fonc.2024.1448336)
Supplement: Supplementary file 15 [file Table3.doc]

**Table S3 Methodological quality assessments (Jadad scale) of the included studies.**

| **Study** | | **Randomization** | **Masking** | **Accountability of all patients** | **Quality (score)** |
| --- | --- | --- | --- | --- | --- |
| NCT04035486(FLAURA2) | Janne 2024 [9],Planchard 2023 [18] | ** | ** | * | 5 |
| NCT01951469(GAP BRAIN) | Hou 2023 [8] | ** | ** | * | 5 |
| UMIN000006340(NEJ009) | Miyauchi 2022 [10], Hosomi 2020 [19] | ** | ** | * | 5 |
| NCT02148380 | Lou 2022 [7] | ** | * | * | 4 |
| CTRI/2016/08/007149 | Noronha 2020 [20] | ** | * | * | 4 |
