# Supplementary material for: The benefit and risk of addition of chemotherapy to EGFR tyrosine kinase inhibitors for EGFR-positive non-small cell lung cancer patients with brain metastases: a meta-analysis based on randomized controlled trials
Source: Front Oncol. 2024 Oct 21;14:1448336. doi: 10.3389/fonc.2024.1448336 (PMC11532100; doi:10.3389/fonc.2024.1448336)
Supplement: Supplementary file 16 [file Table4.doc]

**Table S4** GRADE quality assessment by therapeutic strategy and study design for the outcomes.

| **Primary outcomes** | **No. of Participants** | | **Differences (95%CI)^a^** | **Quality Assessment** | | | | | **Quality** |
| --- | --- | --- | --- | --- | --- | --- | --- | --- | --- |
|  | **ETC** | **CT** |  | **Risk of Bias^b^** | **Inconsistency** | **Indirectness** | **Imprecision** | **Publication Bias^c^** |  |
| **Survival** |  |  |  |  |  |  |  |  |  |
| OS | 126 | 138 | 0.64 [0.48, 0.87] | Low | No inconsistency | No indirectness | No imprecision | Unlikely | High |
| PFS | 266 | 269 | 0.42 [0.34, 0.52] | Low | No inconsistency | No indirectness | No imprecision | Unlikely | High |
| CNS-PFS | 198 | 185 | 0.42 [0.31, 0.57] | Low | No inconsistency | No indirectness | No imprecision | Unlikely | High |
| **Subgroup analysis of survival** |  |  |  |  |  |  |  |  |  |
| **OS** |  |  |  |  |  |  |  |  |  |
| All patients | 264 | | 0.64 [0.48, 0.87] | Low | No inconsistency | No indirectness | No imprecision | Unlikely | High |
| Age < 65 years | 127 | | 0.64 [0.40, 1.02] | Low | No inconsistency | No indirectness | No imprecision | Unlikely | High |
| Age > 65 years | 34 | | 1.05 [0.40, 2.75] | Low | No inconsistency | No indirectness | No imprecision | Unlikely | High |
| Sex - Female | 87 | | 0.64 [0.35, 1.17] | Low | No inconsistency | No indirectness | No imprecision | Unlikely | High |
| Sex - Male | 74 | | 0.60 [0.34, 1.07] | Low | No inconsistency | No indirectness | No imprecision | Unlikely | High |
| Smoking status - Smoker | 41 | | 0.77 [0.35, 1.69] | Low | No inconsistency | No indirectness | No imprecision | Unlikely | High |
| Smoking status - Non-smoker | 120 | | 0.56 [0.34, 0.94] | Low | No inconsistency | No indirectness | No imprecision | Unlikely | High |
| ECOG PS - 0 | 43 | | 0.36 [0.13, 0.99] | Low | No inconsistency | No indirectness | No imprecision | Unlikely | High |
| ECOG PS - 1 | 118 | | 0.78 [0.49, 1.24] | Low | No inconsistency | No indirectness | No imprecision | Unlikely | High |
| Large intracranial tumor size - < 20mm | 110 | | 0.53 [0.32, 0.88] | Low | No inconsistency | No indirectness | No imprecision | Unlikely | High |
| Large intracranial tumor size - > 20mm | 51 | | 0.97 [0.46, 2.04] | Low | No inconsistency | No indirectness | No imprecision | Unlikely | High |
| EGFR mutation - Ex19del | 85 | | 0.40 [0.22, 0.73] | Low | No inconsistency | No indirectness | No imprecision | Unlikely | High |
| EGFR mutation - L858R | 70 | | 0.83 [0.45, 1.54] | Low | No inconsistency | No indirectness | No imprecision | Unlikely | High |
| Extracranial metastases - Yes | 118 | | 0.54 [0.33, 0.88] | Low | No inconsistency | No indirectness | No imprecision | Unlikely | High |
| Extracranial metastases - No | 43 | | 0.76 [0.31, 1.88] | Low | No inconsistency | No indirectness | No imprecision | Unlikely | High |
| **PFS** |  |  |  |  |  |  |  |  |  |
| All patients | 535 | | 0.42 [0.34, 0.52] | Low | No inconsistency | No indirectness | No imprecision | Unlikely | High |
| Age < 65 years | 127 | | 0.40 [0.26, 0.62] | Low | No inconsistency | No indirectness | No imprecision | Unlikely | High |
| Age > 65 years | 34 | | 0.42 [0.15, 1.19] | Low | No inconsistency | No indirectness | No imprecision | Unlikely | High |
| Sex - Female | 87 | | 0.33 [0.18, 0.60] | Low | No inconsistency | No indirectness | No imprecision | Unlikely | High |
| Sex - Male | 74 | | 0.45 [0.26, 0.78] | Low | No inconsistency | No indirectness | No imprecision | Unlikely | High |
| Smoking status - Smoker | 41 | | 0.43 [0.20, 0.92] | Low | No inconsistency | No indirectness | No imprecision | Unlikely | High |
| Smoking status - Non-smoker | 120 | | 0.36 [0.22, 0.58] | Low | No inconsistency | No indirectness | No imprecision | Unlikely | High |
| ECOG PS - 0 | 43 | | 0.31 [0.13, 0.73] | Low | No inconsistency | No indirectness | No imprecision | Unlikely | High |
| ECOG PS - 1 | 118 | | 0.42 [0.27, 0.66] | Low | No inconsistency | No indirectness | No imprecision | Unlikely | High |
| EGFR mutation - Ex19del | 85 | | 0.32 [0.18, 0.57] | Low | No inconsistency | No indirectness | No imprecision | Unlikely | High |
| EGFR mutation - L858R | 70 | | 0.43 [0.24, 0.77] | Low | No inconsistency | No indirectness | No imprecision | Unlikely | High |
| Extracranial metastases - Yes | 118 | | 0.39 [0.24, 0.63] | Low | No inconsistency | No indirectness | No imprecision | Unlikely | High |
| Extracranial metastases - No | 43 | | 0.34 [0.15, 0.77] | Low | No inconsistency | No indirectness | No imprecision | Unlikely | High |
| EGFR TKIs - Osimertinib | 222 | | 0.47 [0.33, 0.66] | Low | No inconsistency | No indirectness | No imprecision | Unlikely | High |
| EGFR TKIs - Gefitinib | 313 | | 0.39 [0.30, 0.52] | Low | No inconsistency | No indirectness | No imprecision | Unlikely | High |
| **CNS-PFS** |  |  |  |  |  |  |  |  |  |
| All patients | 383 | | 0.42 [0.31, 0.57] | Low | No inconsistency | No indirectness | No imprecision | Unlikely | High |
| Age < 65 years | 127 | | 0.40 [0.26, 0.61] | Low | No inconsistency | No indirectness | No imprecision | Unlikely | High |
| Age > 65 years | 34 | | 0.21 [0.06, 0.74] | Low | No inconsistency | No indirectness | No imprecision | Unlikely | High |
| Sex - Female | 87 | | 0.28 [0.16, 0.49] | Low | No inconsistency | No indirectness | No imprecision | Unlikely | High |
| Sex - Male | 74 | | 0.43 [0.25, 0.73] | Low | No inconsistency | No indirectness | No imprecision | Unlikely | High |
| Smoking status - Smoker | 41 | | 0.49 [0.24, 1.00] | Low | No inconsistency | No indirectness | No imprecision | Unlikely | High |
| Smoking status - Non-smoker | 120 | | 0.28 [0.18, 0.45] | Low | No inconsistency | No indirectness | No imprecision | Unlikely | High |
| ECOG PS - 0 | 43 | | 0.20 [0.09, 0.46] | Low | No inconsistency | No indirectness | No imprecision | Unlikely | High |
| ECOG PS - 1 | 118 | | 0.43 [0.28, 0.66] | Low | No inconsistency | No indirectness | No imprecision | Unlikely | High |
| Large intracranial tumor size - < 20mm | 110 | | 0.31 [0.19, 0.51] | Low | No inconsistency | No indirectness | No imprecision | Unlikely | High |
| Large intracranial tumor size - > 20mm | 51 | | 0.44 [0.23, 0.86] | Low | No inconsistency | No indirectness | No imprecision | Unlikely | High |
| EGFR mutation - Ex19del | 85 | | 0.29 [0.17, 0.50] | Low | No inconsistency | No indirectness | No imprecision | Unlikely | High |
| EGFR mutation - L858R | 70 | | 0.34 [0.19, 0.62] | Low | No inconsistency | No indirectness | No imprecision | Unlikely | High |
| Extracranial metastases - Yes | 118 | | 0.35 [0.22, 0.55] | Low | No inconsistency | No indirectness | No imprecision | Unlikely | High |
| Extracranial metastases - No | 43 | | 0.30 [0.14, 0.65] | Low | No inconsistency | No indirectness | No imprecision | Unlikely | High |
| EGFR TKIs - Osimertinib | 222 | | 0.58 [0.33, 1.01] | Low | No inconsistency | No indirectness | No imprecision | Unlikely | High |
| EGFR TKIs - Gefitinib | 161 | | 0.36 [0.25, 0.52] | Low | No inconsistency | No indirectness | No imprecision | Unlikely | High |
| **Survival rate** |  |  |  |  |  |  |  |  |  |
| **OSR** |  |  |  |  |  |  |  |  |  |
| OSR-6m | 87/88 | 85/88 | 1.02 [0.97, 1.08] | Low | No inconsistency | No indirectness | No imprecision | Unlikely | High |
| OSR-12m | 85/88 | 76/88 | 1.12 [1.02, 1.23] | Low | No inconsistency | No indirectness | No imprecision | Unlikely | High |
| OSR-18m | 69/88 | 58/88 | 1.56 [0.60, 4.05] | Low | Serious (-1) | No indirectness | No imprecision | Unlikely | Medium |
| OSR-24m | 56/88 | 50/88 | 1.12 [0.88, 1.43] | Low | No inconsistency | No indirectness | No imprecision | Unlikely | High |
| OSR-30m | 46/80 | 38/81 | 1.23 [0.91, 1.65] | Low | No inconsistency | No indirectness | No imprecision | Unlikely | High |
| OSR-36m | 39/80 | 19/81 | 2.08 [1.32, 3.27] | Low | No inconsistency | No indirectness | No imprecision | Unlikely | High |
| OSR-42m | 29/80 | 16/81 | 1.84 [1.08, 3.11] | Low | No inconsistency | No indirectness | No imprecision | Unlikely | High |
| OSR-48m | 19/80 | 11/81 | 1.75 [0.89, 3.44] | Low | No inconsistency | No indirectness | No imprecision | Unlikely | High |
| OSR-54m | 18/80 | 6/81 | 3.04 [1.27, 7.26] | Low | No inconsistency | No indirectness | No imprecision | Unlikely | High |
| OSR-60m | 15/80 | 2/81 | 7.59 [1.79, 32.14] | Low | No inconsistency | No indirectness | No imprecision | Unlikely | High |
| **PFSR** |  |  |  |  |  |  |  |  |  |
| PFSR-6m | 179/198 | 154/185 | 1.09 [0.95, 1.25] | Low | Serious (-1) | No indirectness | No imprecision | Unlikely | Medium |
| PFSR-12m | 155/198 | 88/185 | 1.82 [0.84, 3.96] | Low | Serious (-1) | No indirectness | No imprecision | Unlikely | Medium |
| PFSR-18m | 108/198 | 52/185 | 2.14 [1.08, 4.26] | Low | Serious (-1) | No indirectness | No imprecision | Unlikely | Medium |
| PFSR-24m | 79/198 | 38/185 | 3.99 [0.36, 44.65] | Low | Serious (-1) | No indirectness | No imprecision | Unlikely | Medium |
| PFSR-30m | 53/118 | 22/104 | 2.12 [1.39, 3.24] | Low | No inconsistency | No indirectness | No imprecision | Unlikely | High |
| **CNS-PFSR** |  |  |  |  |  |  |  |  |  |
| CNS-PFSR-6m | 182/198 | 158/185 | 1.09 [0.82, 1.46] | Low | Serious (-1) | No indirectness | No imprecision | Unlikely | Medium |
| CNS-PFSR-12m | 165/198 | 118/185 | 1.64 [0.33, 8.03] | Low | Serious (-1) | No indirectness | No imprecision | Unlikely | Medium |
| CNS-PFSR-18m | 121/198 | 89/185 | 1.71 [0.51, 5.76] | Low | Serious (-1) | No indirectness | No imprecision | Unlikely | Medium |
| CNS-PFSR-24m | 103/198 | 66/185 | 3.78 [0.19, 74.41] | Low | Serious (-1) | No indirectness | No imprecision | Unlikely | Medium |
| CNS-PFSR-30m | 68/118 | 54/104 | 1.11 [0.87, 1.41] | Low | No inconsistency | No indirectness | No imprecision | Unlikely | High |
| **Responses** |  |  |  |  |  |  |  |  |  |
| **Overall responses** |  |  |  |  |  |  |  |  |  |
| ORR | 64/80 | 52/81 | 1.25 [1.02, 1.52] | Low | No inconsistency | No indirectness | No imprecision | Unlikely | High |
| DCR | 76/80 | 77/81 | 1.00 [0.93, 1.07] | Low | No inconsistency | No indirectness | No imprecision | Unlikely | High |
| CR | 0/80 | 0/81 | Not estimable | Low | No inconsistency | No indirectness | No imprecision | Unlikely | High |
| PR | 64/80 | 52/81 | 1.25 [1.02, 1.52] | Low | No inconsistency | No indirectness | No imprecision | Unlikely | High |
| SD | 12/80 | 25/81 | 0.49 [0.26, 0.90] | Low | No inconsistency | No indirectness | No imprecision | Unlikely | High |
| **CNS responses** | / | / |  |  |  |  |  |  |  |
| CNS-ORR | 154/198 | 123/185 | 1.19 [0.93, 1.51] | Low | Serious (-1) | No indirectness | No imprecision | Unlikely | Medium |
| CNS-DCR | 183/198 | 173/185 | 0.99 [0.94, 1.04] | Low | No inconsistency | No indirectness | No imprecision | Unlikely | High |
| CNS-CR | 79/198 | 54/185 | 1.31 [1.02, 1.70] | Low | No inconsistency | No indirectness | No imprecision | Unlikely | High |
| CNS-PR | 75/198 | 69/185 | 0.89 [0.31, 2.52] | Low | Serious (-1) | No indirectness | No imprecision | Unlikely | Medium |
| CNS-SD | 29/198 | 40/185 | 0.67 [0.43, 1.03] | Low | No inconsistency | No indirectness | No imprecision | Unlikely | High |
| **Progression Status** |  |  |  |  |  |  |  |  |  |
| Total progression | 89/198 | 101/185 | 0.85 [0.72, 1.01] | Low | No inconsistency | No indirectness | No imprecision | Unlikely | High |
| CNS progression | 61/198 | 83/185 | 0.72 [0.58, 0.90] | Low | No inconsistency | No indirectness | No imprecision | Unlikely | High |
| CNS progression In existing target CNS lesions | 48/198 | 56/185 | 0.60 [0.17, 2.20] | Low | Serious (-1) | No indirectness | No imprecision | Unlikely | Medium |
| CNS progression In new CNS lesions | 37/198 | 58/185 | 0.63 [0.45, 0.87] | Low | No inconsistency | No indirectness | No imprecision | Unlikely | High |
| **Safety** |  |  |  |  |  |  |  |  |  |
| **Safety summary** |  |  |  |  |  |  |  |  |  |
| Total adverse events | 196/198 | 177/185 | 1.04 [0.96, 1.12] | Low | Serious (-1) | No indirectness | No imprecision | Unlikely | Medium |
| Grade 3-5 adverse events | 107/198 | 47/185 | 2.10 [1.59, 2.77] | Low | No inconsistency | No indirectness | No imprecision | Unlikely | High |
| Serious adverse events | 44/118 | 23/104 | 1.69 [1.10, 2.59] | Low | No inconsistency | No indirectness | No imprecision | Unlikely | High |
| Fatal adverse events | 7/118 | 3/104 | 2.06 [0.55, 7.75] | Low | No inconsistency | No indirectness | No imprecision | Unlikely | High |
| Discontinuation due to adverse events | 56/198 | 6/185 | 7.73 [3.57, 16.77] | Low | No inconsistency | No indirectness | No imprecision | Unlikely | High |
| Dose interruption due to adverse events | 10/80 | 7/81 | 1.45 [0.58, 3.61] | Low | No inconsistency | No indirectness | No imprecision | Unlikely | High |
| Treatment-related adverse events | 112/118 | 92/104 | 1.07 [0.99, 1.16] | Low | No inconsistency | No indirectness | No imprecision | Unlikely | High |
| Grade 3-5 treatment-related adverse events | 58/118 | 14/104 | 3.65 [2.17, 6.15] | Low | No inconsistency | No indirectness | No imprecision | Unlikely | High |
| Serious treatment-related adverse events | 20/118 | 9/104 | 1.96 [0.93, 4.11] | Low | No inconsistency | No indirectness | No imprecision | Unlikely | High |
| Fatal treatment-related adverse events | 3/198 | 0/185 | 3.75 [0.42, 33.49] | Low | No inconsistency | No indirectness | No imprecision | Unlikely | High |
| **Any grade adverse Events** |  |  |  |  |  |  |  |  |  |
| Anorexia | 58/80 | 15/81 | 3.92 [2.43, 6.30] | Low | No inconsistency | No indirectness | No imprecision | Unlikely | High |
| Alanine aminotransferase increase | 56/80 | 42/81 | 1.35 [1.05, 1.74] | Low | No inconsistency | No indirectness | No imprecision | Unlikely | High |
| Leukopenia | 50/80 | 6/81 | 8.44 [3.84, 18.56] | Low | No inconsistency | No indirectness | No imprecision | Unlikely | High |
| Neutropenia | 49/80 | 6/81 | 8.27 [3.75, 18.21] | Low | No inconsistency | No indirectness | No imprecision | Unlikely | High |
| Aspartate aminotransferase increase | 46/80 | 41/81 | 1.14 [0.85, 1.51] | Low | No inconsistency | No indirectness | No imprecision | Unlikely | High |
| Anemia | 45/80 | 25/81 | 1.82 [1.25, 2.66] | Low | No inconsistency | No indirectness | No imprecision | Unlikely | High |
| Alkaline phosphatase increase | 45/80 | 31/81 | 1.47 [1.05, 2.06] | Low | No inconsistency | No indirectness | No imprecision | Unlikely | High |
| Rash | 45/80 | 45/81 | 1.01 [0.77, 1.33] | Low | No inconsistency | No indirectness | No imprecision | Unlikely | High |
| Nausea | 40/80 | 3/81 | 13.50 [4.35, 41.87] | Low | No inconsistency | No indirectness | No imprecision | Unlikely | High |
| Fatigue | 37/80 | 20/81 | 1.87 [1.20, 2.93] | Low | No inconsistency | No indirectness | No imprecision | Unlikely | High |
| Vomiting | 32/80 | 1/81 | 32.40 [4.54, 231.46] | Low | No inconsistency | No indirectness | No imprecision | Unlikely | High |
| Hypoalbuminemia | 30/80 | 21/81 | 1.45 [0.91, 2.30] | Low | No inconsistency | No indirectness | No imprecision | Unlikely | High |
| Pruritus | 26/80 | 29/81 | 0.91 [0.59, 1.40] | Low | No inconsistency | No indirectness | No imprecision | Unlikely | High |
| Blood creatinine increase | 22/80 | 6/81 | 3.71 [1.59, 8.67] | Low | No inconsistency | No indirectness | No imprecision | Unlikely | High |
| Diarrhea | 20/80 | 26/81 | 0.78 [0.48, 1.28] | Low | No inconsistency | No indirectness | No imprecision | Unlikely | High |
| Thrombocytopenia | 19/80 | 2/81 | 9.62 [2.32, 39.95] | Low | No inconsistency | No indirectness | No imprecision | Unlikely | High |
| Hypocalcemia | 19/80 | 13/81 | 1.48 [0.78, 2.79] | Low | No inconsistency | No indirectness | No imprecision | Unlikely | High |
| Constipation | 18/80 | 4/81 | 4.56 [1.61, 12.87] | Low | No inconsistency | No indirectness | No imprecision | Unlikely | High |
| Hypokalemia | 11/80 | 16/81 | 0.70 [0.34, 1.41] | Low | No inconsistency | No indirectness | No imprecision | Unlikely | High |
| Hyponatremia | 9/80 | 13/81 | 0.70 [0.32, 1.55] | Low | No inconsistency | No indirectness | No imprecision | Unlikely | High |
| Blood bilirubin increase | 7/80 | 11/81 | 0.64 [0.26, 1.58] | Low | No inconsistency | No indirectness | No imprecision | Unlikely | High |
| Paronychia | 6/80 | 9/81 | 0.68 [0.25, 1.81] | Low | No inconsistency | No indirectness | No imprecision | Unlikely | High |
| Hypercalcemia | 5/80 | 1/81 | 5.06 [0.60, 42.37] | Low | No inconsistency | No indirectness | No imprecision | Unlikely | High |
| Hyperkalemia | 3/80 | 1/81 | 3.04 [0.32, 28.59] | Low | No inconsistency | No indirectness | No imprecision | Unlikely | High |
| **Grade 3-5 adverse Events** |  |  |  |  |  |  |  |  |  |
| Alanine aminotransferase increase | 9/80 | 12/81 | 0.76 [0.34, 1.70] | Low | No inconsistency | No indirectness | No imprecision | Unlikely | High |
| Neutropenia | 6/80 | 0/81 | 13.16 [0.75, 229.80] | Low | No inconsistency | No indirectness | No imprecision | Unlikely | High |
| Nausea | 6/80 | 0/81 | 13.16 [0.75, 229.80] | Low | No inconsistency | No indirectness | No imprecision | Unlikely | High |
| Anorexia | 4/80 | 0/81 | 9.11 [0.50, 166.51] | Low | No inconsistency | No indirectness | No imprecision | Unlikely | High |
| Diarrhea | 4/80 | 0/81 | 9.11 [0.50, 166.51] | Low | No inconsistency | No indirectness | No imprecision | Unlikely | High |
| Leukopenia | 3/80 | 0/81 | 7.09 [0.37, 135.02] | Low | No inconsistency | No indirectness | No imprecision | Unlikely | High |
| Anemia | 3/80 | 1/81 | 3.04 [0.32, 28.59] | Low | No inconsistency | No indirectness | No imprecision | Unlikely | High |
| Vomiting | 3/80 | 0/81 | 7.09 [0.37, 135.02] | Low | No inconsistency | No indirectness | No imprecision | Unlikely | High |
| Aspartate aminotransferase increase | 2/80 | 6/81 | 0.34 [0.07, 1.62] | Low | No inconsistency | No indirectness | No imprecision | Unlikely | High |
| Fatigue | 2/80 | 0/81 | 5.06 [0.25, 103.80] | Low | No inconsistency | No indirectness | No imprecision | Unlikely | High |
| Rash | 2/80 | 1/81 | 2.02 [0.19, 21.89] | Low | No inconsistency | No indirectness | No imprecision | Unlikely | High |
| Thrombocytopenia | 1/80 | 0/81 | 3.04 [0.13, 73.46] | Low | No inconsistency | No indirectness | No imprecision | Unlikely | High |
| Blood creatinine increase | 1/80 | 1/81 | 1.01 [0.06, 15.91] | Low | No inconsistency | No indirectness | No imprecision | Unlikely | High |
| Pruritus | 1/80 | 0/81 | 3.04 [0.13, 73.46] | Low | No inconsistency | No indirectness | No imprecision | Unlikely | High |
| Paronychia | 1/80 | 0/81 | 3.04 [0.13, 73.46] | Low | No inconsistency | No indirectness | No imprecision | Unlikely | High |
| Alkaline phosphatase increase | 0/80 | 1/81 | 0.34 [0.01, 8.16] | Low | No inconsistency | No indirectness | No imprecision | Unlikely | High |
| Blood bilirubin increase | 0/80 | 1/81 | 0.34 [0.01, 8.16] | Low | No inconsistency | No indirectness | No imprecision | Unlikely | High |

**Abbreviations:** AEs: Adverse effects; CI: Confidence interval; CNS: Central Nervous System; CR: Complete response; ECOG: Eastern Cooperative Oncology Group; EGFR: Epidermal growth factor receptor; ET: EGFR tyrosine kinase inhibitors alone; ETC: EGFR tyrosine kinase inhibitors in combination of chemotherapy; GRADE: Grading of Recommendations, Assessment, Development, and Evaluation; HR: Hazard ratio; ORR: Objective response rate; OS: Overall survival; OSR: Overall survival rate; P: Probability; PFS: Progression-free survival; RCT: randomized controlled trial; RR: Risk ratio; TKIs: Tyrosine kinase inhibitors.

^a^ Differences: hazard ratio (HR) for OS, PFS, and CNS-PFS; risk ratios (RR) for OSR, PFSR, CNS-PFSR, responses, and AEs.

^b^ Risk of bias assessed using the Jadad scale for randomized controlled trials.

^c^ Publication bias was explored through visual inspection of the funnel plots.
