# Supplementary material for: The benefit and risk of addition of chemotherapy to EGFR tyrosine kinase inhibitors for EGFR-positive non-small cell lung cancer patients with brain metastases: a meta-analysis based on randomized controlled trials
Source: Front Oncol. 2024 Oct 21;14:1448336. doi: 10.3389/fonc.2024.1448336 (PMC11532100; doi:10.3389/fonc.2024.1448336)
Supplement: Supplementary file 17 [file Table5.doc]

**Table S5** Grade 3-5 adverse events.

| **Adverse events** | **ETC** | |  | **ET** | | **Risk ratio [95% CI]** | **P** |
| --- | --- | --- | --- | --- | --- | --- | --- |
|  | **Event/total** | **%** |  | **Event/total** | **%** |  |  |
| Alanine aminotransferase increase | 9/80 | 11.25% |  | 12/81 | 14.81% | 0.76 [0.34, 1.70] | 0.50 |
| Neutropenia | 6/80 | 7.50% |  | 0/81 | 0.00% | 13.16 [0.75, 229.80] | 0.08 |
| Nausea | 6/80 | 7.50% |  | 0/81 | 0.00% | 13.16 [0.75, 229.80] | 0.08 |
| Anorexia | 4/80 | 5.00% |  | 0/81 | 0.00% | 9.11 [0.50, 166.51] | 0.14 |
| Diarrhea | 4/80 | 5.00% |  | 0/81 | 0.00% | 9.11 [0.50, 166.51] | 0.14 |
| Leukopenia | 3/80 | 3.75% |  | 0/81 | 0.00% | 7.09 [0.37, 135.02] | 0.19 |
| Anemia | 3/80 | 3.75% |  | 1/81 | 1.23% | 3.04 [0.32, 28.59] | 0.33 |
| Vomiting | 3/80 | 3.75% |  | 0/81 | 0.00% | 7.09 [0.37, 135.02] | 0.19 |
| Aspartate aminotransferase increase | 2/80 | 2.50% |  | 6/81 | 7.41% | 0.34 [0.07, 1.62] | 0.18 |
| Fatigue | 2/80 | 2.50% |  | 0/81 | 0.00% | 5.06 [0.25, 103.80] | 0.29 |
| Rash | 2/80 | 2.50% |  | 1/81 | 1.23% | 2.02 [0.19, 21.89] | 0.56 |
| Thrombocytopenia | 1/80 | 1.25% |  | 0/81 | 0.00% | 3.04 [0.13, 73.46] | 0.49 |
| Blood creatinine increase | 1/80 | 1.25% |  | 1/81 | 1.23% | 1.01 [0.06, 15.91] | 0.99 |
| Pruritus | 1/80 | 1.25% |  | 0/81 | 0.00% | 3.04 [0.13, 73.46] | 0.49 |
| Paronychia | 1/80 | 1.25% |  | 0/81 | 0.00% | 3.04 [0.13, 73.46] | 0.49 |
| Alkaline phosphatase increase | 0/80 | 0.00% |  | 1/81 | 1.23% | 0.34 [0.01, 8.16] | 0.50 |
| Blood bilirubin increase | 0/80 | 0.00% |  | 1/81 | 1.23% | 0.34 [0.01, 8.16] | 0.50 |

**Abbreviations:** CI: Confidence interval; ET: EGFR tyrosine kinase inhibitors alone; ETC: EGFR tyrosine kinase inhibitors in combination of chemotherapy; P: Probability.
