# Supplementary material for: The benefit and risk of addition of chemotherapy to EGFR tyrosine kinase inhibitors for EGFR-positive non-small cell lung cancer patients with brain metastases: a meta-analysis based on randomized controlled trials
Source: Front Oncol. 2024 Oct 21;14:1448336. doi: 10.3389/fonc.2024.1448336 (PMC11532100; doi:10.3389/fonc.2024.1448336)
Supplement: Supplementary file 18 [file Table6.doc]

**Table S6** Prior published meta-analysis regarding the conbination of EGFR-TKI with Chemotherapy.

| **Study** | **Published year** | **Intervention** | **Comparison** | **Treatment line** | **Patients** | **Included study** | **Study design** | **Outcomes assessed** |
| --- | --- | --- | --- | --- | --- | --- | --- | --- |
| Zhou et al. [1] | 2024 | EGFR-TKI + Chemotherapy | EGFR-TKI | Unrestricted | 15887 | 34 | RCT | AEs |
| Landre et al. [2] | 2024 | EGFR-TKI + Chemotherapy | EGFR-TKI | First line | 1413 | 3 | RCT | Survial |
| Yi et al. [3] | 2023 | Gefitinib + Chemotherapy | Gefitinib | Unrestricted | 1528 | 10 | RCT、CT | Survial, Responses, AEs |
| Lei et al. [4] | 2023 | EGFR-TKI + Chemotherapy/Antiangiogenic therapy | Osimertinib | Unrestricted | 2325 | 14 | RCT、CT | Survial, Responses, AEs |
| Dai et al. [5] | 2023 | EGFR-TKI + Chemotherapy | EGFR-TKI + Antiangiogenic therapy | Unrestricted | 1185 | 19 | RCT、CT | Survial, Responses, AEs |
| Cui et al. [6] | 2023 | EGFR-TKI + Chemotherapy | EGFR-TKI | Adjuvant treatment | 1334 | 10 | RCT、CT | Survial, Responses, AEs |
| Wang et al. [7] | 2022 | Gefitinib + Chemotherapy | Gefitinib | First line | 1014 | 5 | RCT | Survial, Responses |
| Zhu et al. [8] | 2021 | EGFR-TKI + Chemotherapy | EGFR-TKI | Unrestricted | 1354 | 10 | RCT | Survial, Responses |
| Chen et al. [9] | 2021 | EGFR-TKI + Chemotherapy/Antiangiogenic therapy | EGFR-TKI/Chemotherapy | First line | 2367 | 10 | RCT、CT | Survial, Responses, AEs |
| Zhang et al. [10] | 2016 | EGFR-TKI + Chemotherapy | EGFR-TKI/Chemotherapy | Unrestricted | 5861 | 15 | RCT、CT | Survial, Responses, AEs |
| Hong et al. [11] | 2016 | EGFR-TKI + Chemotherapy | Chemotherapy | First line | 933 | 6 | RCT | Survial, Responses, AEs |
| Yan et al. [12] | 2015 | EGFR-TKI + Chemotherapy | Chemotherapy | Unrestricted | 1660 | 10 | RCT | Survial, Responses, AEs |
| Yan et al. [13] | 2015 | EGFR-TKI + Chemotherapy | EGFR-TKI/Chemotherapy | First line | 4675 | 6 | RCT | Survial, Responses, AEs |
| Xu et al. [14] | 2015 | Erlotinib + Chemotherapy | Chemotherapy | Unrestricted | 3599 | 9 | RCT | Survial, Responses |
| Xiao et al. [15] | 2015 | EGFR-TKI + Chemotherapy | EGFR-TKI/Chemotherapy | First line | 1168 | 7 | RCT、CT | Survial, Responses,AEs |
| Sheng et al. [16] | 2015 | EGFR-TKI + Chemotherapy | EGFR-TKI | First line | 2160 | 12 | RCT | Survial, Responses |
| OuYang et al. [17] | 2013 | EGFR-TKI + Chemotherapy | EGFR-TKI/Chemotherapy | First line | 4585 | 8 | RCT | Survial, Responses,AEs |

**Abbreviations:** AEs: Adverse effects; CT: Cohort study; EGFR: Epidermal growth factor receptor; NSCLC: Non-small cell lung cancer; RCT: randomized controlled trial;TKIs: Tyrosine kinase inhibitors.

**Supplementary references:**

1. Zhou S, Kishi N, Alerasool P, Rohs NC. Adverse Event Profile of Epidermal Growth Factor Receptor Tyrosine Kinase Inhibitors for Non-small Cell Lung Cancer: An Updated Meta-analysis. Target Oncol. 2024;19(4):547-564.

2. Landre T, Assié JB, Chouahnia K, Des Guetz G, Auliac JB, Chouaïd C. First-line concomitant EGFR-TKI + chemotherapy versus EGFR-TKI alone for advanced EGFR-mutated NSCLC: a meta-analysis of randomized phase III trials. Expert Rev Anticancer Ther. 2024;24(8):775-780.

3. Yi M, He T, Wang K, Wei Y. Comparison of gefitinib plus chemotherapy versus gefitinib alone for advanced non‑small‑cell lung cancer: A meta analysis. Clinics (Sao Paulo). 2023;78:100152.

4. Lei Y, Duan J, Zhang Q, Li Q. Comparation of EGFR-TKI (EGFR tyrosine kinase inhibitors) combination therapy and osimertinib for untreated EGFR-mutated advanced non-small cell lung cancers: A systematic review and network meta-analysis. Medicine (Baltimore). 2023;102(30):e34483.

5. Dai J, Liu X, Li J, Qu T, Cui Y, Jin S, et al. Efficacy and safety of antiangiogenic agents or chemotherapy plus EGFR-TKIs in advanced non-small cell lung cancer: A systematic review and network meta-analysis. Thorac Cancer. 202314(6):535-543.

6. Cui X, Li X, Lv C, Yan S, Wang J, Wu N. Efficacy and safety of adjuvant EGFR TKI alone and in combination with chemotherapy for resected EGFR mutation-positive non-small cell lung cancer: A Bayesian network meta-analysis. Crit Rev Oncol Hematol. 2023;186:104010.

7. Wang BC, Zhang WX, Kuang BH, Lin GH. The efficacy and tolerability of combining pemetrexed-based chemotherapy with gefitinib in the first-line treatment of non-small cell lung cancer with mutated EGFR: A pooled analysis of randomized clinical trials. PLoS One. 2022;17(10):e0275919.

8. Zhu CM, Lian XY, Zhang HY, Bai L, Yun WJ, Zhao RH, et al. EGFR tyrosine kinase inhibitors alone or in combination with chemotherapy for non-small-cell lung cancer with EGFR mutations: A meta-analysis of randomized controlled trials. J Cancer Res Ther. 2021;17(3):664-670.

9. Chen Y, Wen S, Wu Y, Shi L, Xu X, Shen B. Efficacy and safety of first-generation epidermal growth factor receptor (EGFR) tyrosine kinase inhibitors (TKIs) combined with chemotherapy or antiangiogenic therapy as first-line treatment in patients with EGFR-mutant non-small cell lung cancer: A systematic review and meta-analysis. Crit Rev Oncol Hematol. 2021;163:103393.

10. Zhang M, Guo H, Zhao S, Wang Y, Yang M, Yu J, et al. Efficacy of epidermal growth factor receptor inhibitors in combination with chemotherapy in advanced non-small cell lung cancer: a meta-analysis of randomized controlled trials. Oncotarget. 2016;7(26):39823-39833.

11. Hong C, Mei T, Wang J. Intercalated Combination of Chemotherapy and EGFR-TKIs versus Chemotherapy Alone in the First-line Treatment of Advanced Non-small Cell Lung Cancer: A Meta-analysis. Zhongguo Fei Ai Za Zhi. 2016;19(12):837-846.

12. Yan H, Li Q, Wang W, Zhen H, Cao B. Systems assessment of intercalated combination of chemotherapy and EGFR TKIs versus chemotherapy or EGFR TKIs alone in advanced NSCLC patients. Sci Rep. 2015;5:15355.

13. Yan H, Li H, Li Q, Zhao P, Wang W, Cao B. The Efficacy of Synchronous Combination of Chemotherapy and EGFR TKIs for the First-Line Treatment of NSCLC: A Systematic Analysis. PLoS One. 2015;10(8):e0135829.

14. Xu JL, Jin B, Ren ZH, Lou YQ, Zhou ZR, Yang QZ, et al. Chemotherapy plus Erlotinib versus Chemotherapy Alone for Treating Advanced Non-Small Cell Lung Cancer: A Meta-Analysis. PLoS One. 2015;10(7):e0131278.

15. Xiao BK, Yang JY, Dong JX, Ji ZS, Si HY, Wang WL, et al. Meta-analysis of seven randomized control trials to assess the efficacy and toxicity of combining EGFR-TKI with chemotherapy for patients with advanced NSCLC who failed first-line treatment. Asian Pac J Cancer Prev. 2015;16(7):2915-21.

16. Sheng Z, Zhang Y. EGFR-TKIs combined with chemotherapy versus EGFR-TKIs single agent as first-line treatment for molecularly selected patients with non-small cell lung cancer. Med Oncol. 2015;32(1):420.

17. OuYang PY, Su Z, Mao YP, Deng W, Xie FY. Combination of EGFR-TKIs and chemotherapy as first-line therapy for advanced NSCLC: a meta-analysis. PLoS One. 2013;8(11):e79000.
